# Environment or genetic isolation? An atypical intestinal microbiota in the Maltese honey bee *Apis mellifera* spp. *ruttneri*

**DOI:** 10.3389/fmicb.2023.1127717

**Published:** 2023-02-23

**Authors:** Francesca Gaggìa, Rasmus Riemer Jakobsen, Daniele Alberoni, Loredana Baffoni, Simone Cutajar, David Mifsud, Dennis Sandris Nielsen, Diana Di Gioia

**Affiliations:** ^1^Department of Agricultural and Food Sciences, University of Bologna, Bologna, Italy; ^2^Section of Microbiology and Fermentation, Department of Food Science, Faculty of Science, University of Copenhagen, Copenhagen, Denmark; ^3^Institute of Earth Systems, L-Università tà Malta, Msida, Malta

**Keywords:** honey bees, microbiome, *Bartonella*, *Lactobacillus*, environment, *Apis mellifera* spp. *ruttneri*, *Bombella apis*, mitochondrial haplotype

## Abstract

**Introduction:**

*Apis mellifera* evolved mainly in African, Asian, and European continents over thousands of years, leading to the selection of a considerable number of honey bees subspecies that have adapted to various environments such as hot semi-desert zones and cold temperate zones. With the evolution of honey bee subspecies, it is possible that environmental conditions, food sources, and microbial communities typical of the colonized areas have shaped the honey bee gut microbiota.

**Methods:**

In this study the microbiota of two distinct lineages (mitochondrial haplotypes) of bees *Apis mellifera ruttneri* (lineage A) and *Apis mellifera ligustica* and *carnica* (both lineage C) were compared. Honey bee guts were collected in a dry period in the respective breeding areas (the island of Malta and the regions of Emilia-Romagna and South Tyrol in Italy). Microbial DNA from the honey bee gut was extracted and amplified for the V3-V4 regions of the 16S rRNA gene for bacteria and for ITS2 for fungi.

**Results:**

The analyses carried out show that the Maltese lineage A honey bees have a distinctive microbiota when compared to Italian lineage C honey bees, with the most abundant genera being Bartonellaceae and Lactobacillaceae, respectively. Lactobacillaceae in Maltese Lineage A honey bees consist mainly of *Apilactobacillus* instead of *Lactobacillus* and *Bombilactobacillus* in the lineage C. Lineage A honey bee gut microbiota also harbors higher proportions of *Arsenophonus*, *Bombella*, *Commensalibacter*, and *Pseudomonas* when compared to lineage C.

**Discussion:**

The environment seems to be the main driver in the acquisition of these marked differences in the gut microbiota. However, the influence of other factors such as host genetics, seasonality or geography may still play a significant role in the microbiome shaping, in synergy with the environmental aspects.

## Introduction

A new subspecies of honey bees, *Apis mellifera* subsp. *ruttneri*, was identified 25 years ago by Sheppard et al. (1997) in the Maltese Islands. It belongs to the African bee subgroup and is classified close to *Apis mellifera* subsp *intermissa, Apis mellifera* subsp *siciliana* and is distantly related to the European subspecies, as revealed by the morphometric analysis and the mitochondrial haplotype of the tRNAleu-Cox2 region (Zammit-Mangion et al., 2017). The Maltese honey bee shows peculiar characteristics of adaptation to drought as well as very hot and windy weather. It is slightly smaller in size, dark in color with no apparent yellow bands, highly active and resistant to varroosis (Sheppard et al., 1997). These characteristics have developed after thousands of years of isolation on the Maltese Islands.

Honey bees have been classified into five main lineages discriminated according to the mitochondrial haplotype used to characterize evolutionary diversity between and within populations: (a) lineage A (Africa) to which *A. mellifera ruttneri* belongs; (b) lineage Y (Yemen and Ethiopia); (c) lineage O (Oriental, from Turkey to Kazakhstan); (d) lineage C (Carnica, from Central/South Europe) to which *A. mellifera ligustica* and *carnica* belong, and (e) lineage M (Mellifera, from West/North Europe) which comprises over 28 different subspecies, with many others expected to be discovered (Miguel et al., 2007). Lineages have also been divided into subcategories and *A. mellifera ruttneri*, at present, belongs to the mitochondrial sub-haplotypes A4, A8, and A9 (Zammit-Mangion et al., 2017).

Described honey bee subspecies have shown behavioral and morphological adaptations to their native environments, allowing them to better exploit available food resources. Considering how crucial the gut microbiota is for food exploitation in bee nutrition, it is hypothesized that environment, behavior and food quality shapes the microbial community composition at honey bee subspecies level. In fact, recent studies demonstrated how seasonality, landscape (environment and nutrient availability) and host genetic background can impact the microbial profile of different caste of honey bees (Mattila et al., 2012; Kešnerová et al., 2020; Wang et al., 2020).

The main available studies report that the honey bee gut harbors a simple microbial community (Martinson et al., 2011) composed of a limited number of core bacterial species (Sabree et al., 2012), which include both Gram negative and Gram positive groups (Moran, 2015). These bacteria are specific to the bee gut and can be directly transmitted among individuals through social interactions (Zheng et al., 2018). The honey bee gut microbial community is relatively stable over time and space, unless honey bees are subjected to anthropogenic pressures such as the use of antibiotics (Raymann et al., 2017; Baffoni et al., 2021) and/or pesticide treatments in agricultural practices, including glyphosate (Motta et al., 2018) and neonicotinoids (Alberoni et al., 2021a). These studies have generally only addressed the domesticated *A. mellifera* and as such, a description of gut microbial profiles looking at honey bee subspecies have never been convincingly reported. Some studies have regarded the characterization of cultivable lactic acid bacteria and bifidobacteria in different *A. mellifera* subspecies, e.g., *scutellata, mellifera*, and *monticola* (Olofsson et al., 2011), revealing that all share the same Lactobacillaceae and *Bifidobacterium* phylotypes. Sharifpour et al. (2016) isolated and characterized lactic acid bacteria and bifidobacteria from the gut of *A. mellifera* subspecies of West Azerbaijan showing that there is low sequence divergence in comparison with other lactic acid bacteria.

Given the huge interest in honey bee gut microbiota and the relevant papers published on the European *A. mellifera*, this study investigates the gut microbiota of *A. mellifera ruttneri* (lineage A), looking at its core composition and abundance. High throughput sequencing gave an overview of the overall abundance of bacteria and yeast communities; moreover, investigation of the lactobacilli population was also performed with culture-dependent techniques and PCR-DGGE. Data based on the 16S rRNA gene sequencing were used for comparative analysis with data obtained from *A. mellifera* subsp. *ligustica* and *carnica* (lineage C) and for metagenome functional prediction.

To the best of our knowledge, this is the first deep analysis of the Maltese honey bee gut microbiota. The study investigates whether there are distinctive differences in the gut microbiota of the honey bees prevalent in Italy (*A. mellifera ligustica* and *carnica*) and *A. mellifera ruttneri*, since these subspecies have been sampled from niches with different climate conditions and possibility of exchange of genetic resources, in addition to their different mitochondrial haplotypes (C and A) and consequent different phylogenesis. To date, the Maltese honey bee is considered an endangered subspecies due to the importation of different honey bees from the European continent, thus representing a threat to the one hundred pure beehives still present on the Maltese Islands (Jansen, 2018). The investigation was carried out in Malta in three different apiaries with different beekeeping management practices. In one of the test apiaries, the Maltese honey bee is still being reared in terracotta hives called “Migbha,” dating back to Punic times (Supplementary Figures 1A, B), a unique case in Europe.

## Materials and methods

### Sampling location and samples collection

Guts from *Apis mellifera ruttneri* were sampled from three different apiaries located in Malta during April 2016. Sampled honey bees, picked off the brood surface, were between 15–20 days old. The apiary in Għargħur (GH) had been established for more than 80 years as it belongs to a beekeeping family who still rear some of their colonies in terracotta hives, a practice unique to the Maltese Islands and other southern European countries (Supplementary Figure 1). This apiary is located in an urban location (35° 92′22.58″ N, 14° 45′39.58″ E) overlooking a small valley system. The apiary Campus Msida (CM) is located on the University of Malta grounds (35° 90′40.36″ N, 14° 48′33.56″ E) in Wied Għollieqa (Valley) and represents a recently established apiary with around 20 colonies of bees. The environment surrounding CM is best described as abundant agricultural land now dominated by carob trees (*Ceratonia siliqua*) and prickly pear (*Opuntia ficus-indica*). The apiary in Żejtun (ZT) is located at the outskirts of the village (35° 85′98.35″ N, 14° 53′74.71″ E), in an agricultural dwelling where occasional use of pesticides is practised. The main crops cultivated in the area include potatoes, tomatoes and courgettes. For bacteria isolation, a pool of 20 honey bee guts per sampling location were smashed and mixed. Following this, 0.5 mg of each pool was mixed with 4.5 ml of sterilized glycerol broth (meat extract 2.7 g/L, peptone 4.5 g/L, glycerol 100 ml/L) and 1:10 serial dilutions were carried out. For metagenomic analysis, 20 individual guts (both midgut and hindgut) were sampled from each apiary. All samples were immediately shipped on dry ice to the University of Bologna, Italy.

For comparative analysis, data obtained from *Apis mellifera* lineage C were used, samples of both subspecies *ligustica* and *carnica*. The *ligustica* data referred to samples collected in the Emilia-Romagna region (Italy) at Valsamoggia (Bologna, 44°29′45.3″N 11°06′10.4″E) and San Lazzaro di Savena (Bologna, 44°27′28.2″N 11°23′45.8″E) (Alberoni et al., 2021a,b; Baffoni et al., 2021), whereas the *carnica* data referred to samples previously collected in the South Tyrol region, Bolzano (46°22′47.7″N 11°14′14.6″E) (Baffoni et al., 2021). The full list of samples deriving from these studies can be found in Supplementary Table 1.

### DNA extraction, 16S rRNA gene, and ITS library preparation

Genomic DNA from honey bee gut samples was extracted from 20 single honey bee guts per site with the Quick-DNA™ Insect Microbe Miniprep Kit-Zymo Research (ZYMO, Irvine, CA, USA), according to the manufacturer’s instructions. DNA concentration and purity were analyzed with Tecan Infinite 200 PRO reader (Tecan Group Ltd., Mannedorf, Switzerland). DNA was then stored at −20°C. The microbial gut community was determined using tag-encoded 16S rRNA gene MiSeq-based (Illumina, San Diego, CA, USA) high throughput sequencing for bacteria and the variable internal transcribed spacer (ITS)-2 rDNA region for yeast and fungi. The bacterial (V3-V4) and eukaryotic (ITS2) sequencing libraries were prepared according to Takahashi et al. (2014) and Haastrup et al. (2018), respectively. The amplified fragments with adapters and tags were purified and normalized using custom-made beads, pooled and subjected to 250 bp pair-ended MiSeq sequencing. Of the 60 Maltese honey bee guts individually extracted, 30 samples (10 samples from each Maltese testing apiary) were run on a Next Generation Sequencing (NGS) Illumina MiSeq platform for bacterial (V3-V4) sequencing, while the remaining 30 samples were processed for eukaryotic (ITS2) sequencing. The raw dataset containing pair-ended reads with corresponding quality scores were merged and trimmed using Trimmomatic v 0.39 with the following settings, -fastq_minovlen 100, -fastq_maxee 2.0, -fastq_truncal 4, and -fastq_minlen of 160 bp. De-replicating, purging from chimeric reads, and constructing *de novo* zero-radius Operational Taxonomic Units (zASV) were conducted using the UNOISE pipeline Edgar (2018) and taxonomically assigned with –sintex Edgar (2016) coupled to the EZtaxon (Kim et al., 2012) for 16S rRNA gene and UNITE (Kõljalg et al., 2013) for ITS2 as references. A total of 1,25 million reads were obtained for both 16S rRNA genes sequencing. Following assembling and quality filtering (low quality reads, chimeric sequences and unaligned sequences), with an average of 42 thousand sequences per sample. One sample, GH7, failed the sequencing and was therefore removed. The ASVs assigned were 5,513.

### Lactobacilli isolation and identification

For lactic acid bacteria enumeration, serial dilutions were prepared and plated on man rogosa sharpe (MRS) agar (VWR, Milano, Italy) containing 0.01% l-Cysteine-HCl (Merck, Darmstadt, Germany), 0.1% fructose (Sigma-Aldrich, Milano, Italy) and 0.1% cycloheximide (Sigma-Aldrich, Milano, Italy). Analyses were performed in triplicate. Plates were incubated anaerobically at 35°C for 72–120 h, the number of colony forming units (CFU) were recorded and counts were made. Around 100 isolated colonies were re-streaked and purified. For long term storage, purified isolates were stored at −80°C with their respective liquid medium containing 20% glycerol. DNA extraction from pure cultures was performed with the Wizard^®^ Genomic DNA Purification Kit (Promega, Madison, WI, USA). Fingerprinting was then obtained using BOX-PCR, as in Gaggìa et al. (2015). Cluster analysis and grouping BOX profiles was carried out with Bionumerics 7.1 (Applied Maths, Sint-Martens-Latem, Belgium) using Dice’s Coefficient of similarity and the un-weighted pair group method arithmetic averages clustering algorithm (UPGMA). Based on the genotypic grouping, representative isolates were selected, the 16S rRNA gene amplified with primers 8-fw and 1520-rev and sequenced according to Gaggìa et al. (2015). Sequences were then deposited to GenBank^®^^1^ with the following accession number: MT381710-MT381736 and MG649988-MG650060. The obtained 16S rRNA gene sequences were used to generate a phylogenetic tree together with sequences of *A. kunkeei* retrieved from the National Center for Biotechnology Information (NCBI) Genomes RefSeq database (Supplementary Table 2) especially from Germany, Sweden (Tamarit et al., 2015), and Switzerland (Crovadore et al., 2021). The phylogenetic tree was generated with MEGA11 (Tamura et al., 2021) inferred by using the Maximum Likelihood method (K2 + G substitution model) with rate variation among sites. *Lactobacillus melliventris* MT53, *Lactobacillus apis* MT61, and *Gilliamella apicola* MT1 and MT6 were used as outgroups.

### PCR-DGGE analysis of lactobacilli population

PCR-DGGE analyses were performed to investigate lactobacilli populations; for each sampling location, 17 (out of 20) DNA extracted from individual guts were processed. The PCR and subsequent denaturing gradient gel electrophoresis (DGGE) analysis, using the Dcode Universal Mutation Detection System (Bio-Rad Laboratories, Hercules, CA, USA), were performed as described by Alberoni et al. (2018). Denaturing gradient was established at 35–65%. Fingerprinting analyses were carried out using the Bionumerics v 7.1 (Applied Maths, St. Martens-Latem, Belgium) and the UPGMA algorithm based on the Pearson correlation coefficient with an optimization of 1% was applied. Microbial diversity was analyzed with the following parameters: Shannon–Wiener index (H), Simpson index (S), and band evenness (EH), calculated according to Hill et al. (2003). Moreover, principal components analysis (PCA) was carried out by using Bionumerics. Relevant bands were excised from the gels and processed to achieve purified amplicons to be sequenced (Gaggìa et al., 2015). Sequencing was carried out by Eurofins Genomics (Ebersberg, Germany) and obtained sequences were assigned to bacterial species using megablast algorithm.^2^

### Statistical analysis

Bioinformatic analysis was performed using R open-source statistical software v 4.2.1 (R Core Team, 2022) with phyloseq (McMurdie and Holmes, 2013), metagenomeSeq (metagenomeSeq: Statistical analysis for sparse high-throughput sequencing, Paulson, 2014), vegan (Dixon, 2003), ggpubr v 0.4.0 (Kassambara and Kassambara, 2020), and ggplot2 v 3.5.5 (Wickham, 2011) packages. Raw reads were filtered and low-abundance ASVs (below 0.5%) were removed across all samples. The sequencing depth was, on average, 40,103 reads per sample for 16S amplicons and 92,456 for ITS amplicons before filtering. After filtering, 36,109 and 84,396 sequencing were, respectively obtained. For diversity analysis, all samples were rarefied to mean-read depth and cumulative sum scaling (CSS) normalization was used for beta diversity analysis. PICRUSt 2.0 (Phylogenetic Investigation of Communities by Reconstruction of Unobserved States, Douglas et al., 2020) was used to predict functional abundances based on 16S amplicon sequences. Comparisons of alpha diversity was performed using analysis of variance (ANOVA) with Tukey Honest Significant Differences (Tukey HSD) multiple testing correction. Permutational ANOVA (PERMANOVA) was used to evaluate group comparisons of bacterial community composition, using the Bonferroni–Holm method for multiple testing correction. Statistical significance was determined at *p* < 0.05. LEfSe analysis on microbiome data was performed comparing the sampling sites using Galaxy (Blankenberg et al., 2011).

### Climate data elaboration

The monthly climatic data for precipitations (cumulative millimeters of rainfall), average minimum and maximum temperatures, and absolute lower temperatures were retrieved from local repositories. Data from Malta were obtained from the local international Airport,^3^ approximately midpoint of all samplings carried out in the apiaries of Għargħur–GH, Wied Għollieqa–GH, and Campus Msida–CM. The climatic data of the Sud Tirol province (Apiary of Bozen–BZ) were retrieved from the “Südtirol Open Data Alto Adige,”^4^ whereas the climatic data of the Emilia-Romagna region (Municipalities of Valsamoggia–VS) and (San Lazzaro di Savena–SLS) were retrieved from Agenzia regionale per la prevenzione e l’ambiente dell’Emilia Romagna (ARPAE) Emilia-Romagna Environmental Agency database (Dext3r Platform),^5^ using as midpoint of the locality of Zola Predosa (approximately equidistant from the two sampling points), as no data were available for VS and SLS. Retrieved data were used to generate Walter and Lieth climate diagrams (improved Bagnouls and Gaussen climate diagram) of the three main sampling areas. Moreover, to better understand the climatic trend, data from the year prior to sampling were also analyzed. Walter and Lieth climate diagrams were generated with the R statistic package “climatol” (Guijarro and Guijarro, 2019).

## Results

### Results on 16S rRNA gene sequencing on *Apis mellifera ruttneri* gut bacterial communities

Overall, at phylum level, the most representative members were α-proteobacteria (41.70%), γ-proteobacteria (26.70%), Firmicutes (15.60%), β-proteobacteria (7.50%), and Actinobacteria (5.70%) (Supplementary Figure 2), these accounted for 97.30% of the total reads. Supplementary Figures 3, 4 also report the relative abundances at Order and Class level. Among α-proteobacteria, the most representative family was Bartonellaceae accounting for 32.50%, followed by Acetobacteraceae (8.10%). Within γ-proteobacteria, Orbaceae (13.60%), and Morganellaceae (8.80%) were the most abundant families. Finally, Firmicutes, β-proteobacteria, and Actinobacteria, mostly corresponded to the Lactobacillaceae, Neisseriaceae, and the Bifidobacteriaceae families, respectively (Supplementary Figure 5).

**FIGURE 1:**
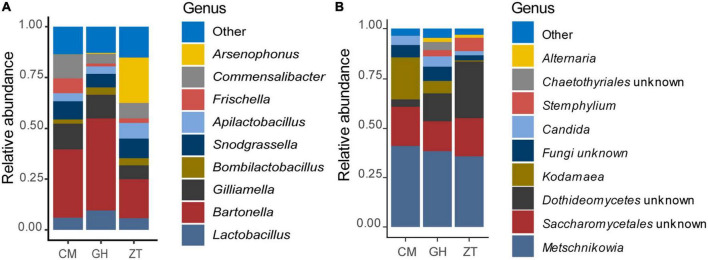
Relative abundance of the gut bacterial **(A)** and fungal **(B)** populations determined by NGS. Bar charts are reporting the major microbial genera cumulated by sampling site in Malta: CM, University of Malta–Campus Msida; GH, Għargħur; ZT, Żejtun.

**FIGURE 2:**
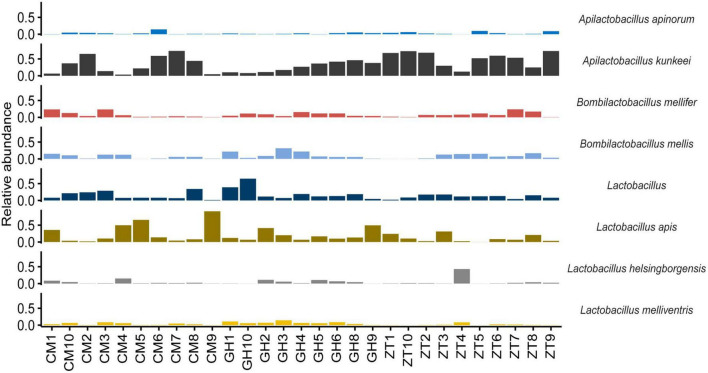
Bar chart showing the relative abundance of the major species belonging to the family Lactobacillaceae in every sampled honey bee gut in Malta determined by NGS.

At genus level (Figure 1 and Supplementary Figure 6), 32.50% of the assigned reads could be ascribed to *Bartonella*, 9.90% to *Arsenophonus*, followed by 9.30% to *Lactobacillus*, 7.40% to *Snodgrassella*, 5.90% to *Commensalibacter*, and 5.50% to *Bifidobacterium*. Less abundant genera were *Apilactobacillus*, *Bombella*, *Bombilactobacillus*, *Pseudomonas*, *Spiroplasma*, and *Acinetobacter* (1–3.00%). In addition, within the Orbaceae family, 8.80% was assigned to *Gilliamella* and 3.70% to *Frischella*. ASVs species assignment among Lactobacillaceae (Figure 2) allowed the detection of the following genera and species: *Lactobacillus apis* 19.55%, *Lactobacillus kimbladii* 13.79%, *Lactobacillus helsingborgensis* 4.46%, and *Lactobacillus melliventris* 3.74%. 15.55% of *Lactobacillus* remained unassigned. The *Apilactobacillus* genus showed up as only two species: *Apilactobacillus apinorum* 3.25% and *Apilactobacillus kunkeei* 23.00%. Within the *Bombilactobacillus* genus, the species *Bombilactobacillus mellis* 8.54% and *Bombilactobacillus mellifer* 8.10% were identified. Interestingly, within *Bartonella*, only 3.80% of ASVs was taxonomically identified as *Bartonella apis*, while the majority of them (96.20%) remained unassigned at species level. Comparing the three Maltese sampling sites (CM, ZT, and GH), the core microbial composition of sampled honey bees did not show appreciable variation in composition.

### Results on ITS gene sequencing on *Apis mellifera ruttneri* gut yeasts community

**FIGURE 3:**
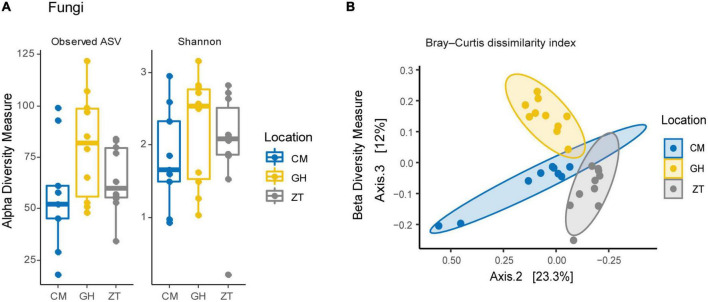
**(A)** Fungal α-diversity within the three sampling sites in Malta: CM, University of Malta–Campus Msida; GH, Għargħur; ZT, Żejtun. **(B)** β-diversity Bray–Curtis dissimilarity index per sampling site on the yeasts microbial community in Malta.

Results for the fungal gut community of the Maltese honey bee revealed the phylum Ascomycota to be, by far, the most abundant, making up 87.26% of the total reads. Basidiomycota counted only 1.53% of the total reads and about 11.20% of the reads remained unassigned at phylum level. The most abundant orders in the Ascomycota phylum were Saccharomycetales and Pleosporales, respectively 65.60 and 4.09%. Saccharomycetales comprised the family Metschnikowiaceae (45.74%–Supplementary Figure 7), followed by unclassified Saccharomycetales family (17.54%). Pleosporales’ most representative family was Pleosporaceae (3.23%). Metschnikowiaceae, at genus level, was represented by *Kodamaea* (8.10%) (with only a specie identified, *Kodamaea ohmeri*) and *Metschnikowia* (34.57%), comprising mostly unidentified species together with *Metschnikowia cibodasensis* (2.19%) and *Metschnikowia chrysoperlae* (0.13%). Pleosporaceae was accounted by *Stemphylium* and *Alternaria* at 2.83 and 0.93%, respectively. Members of the *Candida* genus (assigned to *Saccharomycetales incertae sedis*) accounted for up to 3.33% of the relative abundance, although the relevance of this genus was low amongst samples. The detected species were *C. versatilis* (1.82%), *C. primensis* (0.70%), and *C. kofuensis* (0.52%). Relative abundances, at genus level, are shown in Figure 1B and Supplementary Figure 8. No significative differences were detected in within-sample eukaryotic microbial diversity for neither Shannon and Observed ASVs α-diversity indexes (Figure 3A), whereas between-group comparisons of community composition using Bray–Curtis dissimilarity index (Figure 3B) and Sorensen–Dice indexes for β-diversity showed significant differences between all three Maltese localities (*p* ≤ 0.001 for all comparisons).

### Comparison of the bacterial communities of honey bees sampled in Malta (lineage A) and Italian honey bees (lineage C)

**FIGURE 4:**
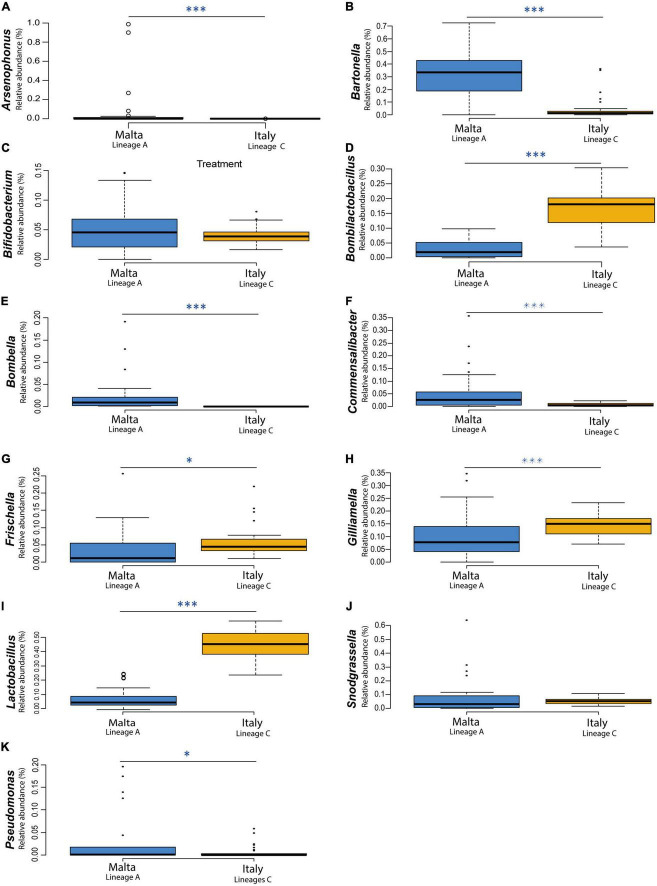
The boxplot chart shows the relative abundance of the gut bacterial populations determined by NGS of the 11 major microbial taxa populating the sampled honey bee guts: **(A)** *Acinetobacter*, **(B)** *Arsenophonus*, **(C)** *Bartonella*, **(D)** *Bifidobacterium*, **(E)** *Bombella*, **(F)** *Commensalibacter*, **(G)** *Frischella*, **(H)** *Gilliamella*, **(I)** *Lactobacillus*, **(J)** *Snodgrassella*, and **(K)** *Pseudomonas* compared for mitochondrial haplotypes. Sampled honey bees mitochondrial haplotype were “A” for the Maltese honey bees and “C” for the Italian honey bees. The box plots compares the average relative abundance values at genus level of 30 sampled honey bees in Malta (Campus Msida, Gharghur, Żeitun) with 30 sampled honey bees in Italy (Bozen, San Lazzaro di Savena, Valsamoggia). Asterisks indicate statistically significant differences (*p < 0.05; ^***^p < 0.01).

**FIGURE 5:**
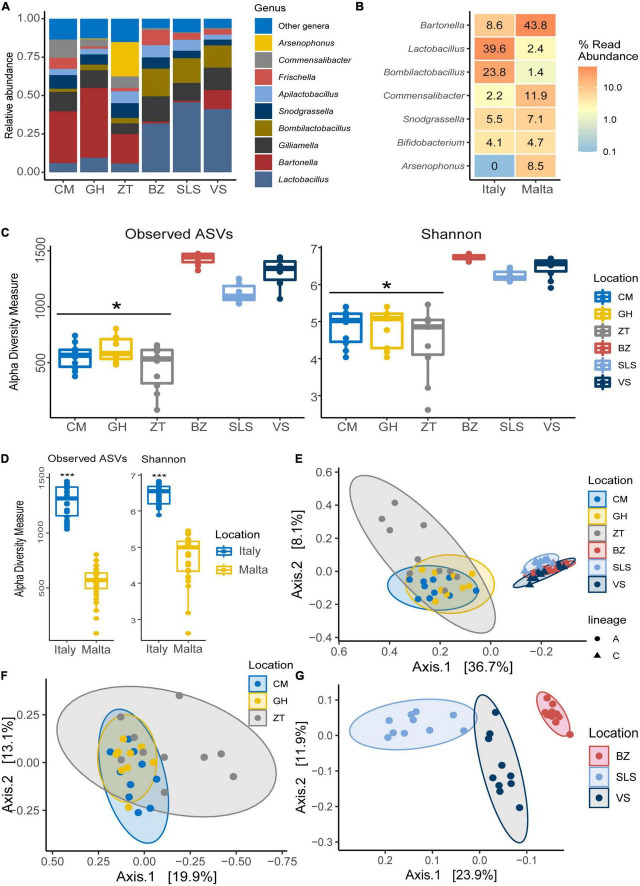
**(A)** Bar chart showing the relative abundance of the major microbial genera in both Malta and Italy determined by NGS. **(B)** Differential abundance heatmap highlighting significantly differentially abundant (*p* < 0.05) microbial genera between honey bees sampled in Italy and in Malta, showing the relative abundance of the genus. **(C)** Boxplot of α-diversity indexes for Observed ASVs and Shannon indexes per sampling site in Italy and Malta. **(D)** Boxplot of α-diversity indexes for Observed ASVs and Shannon indexes per nation (Italy and Malta). **(E)** β-diversity Bray–Curtis dissimilarity index per mitochondrial haplotype (lineage) and sampling site. **(F)** β-Diversity Bray–Curtis dissimilarity index per sampling site in Malta. **(G)** β-diversity Bray–Curtis dissimilarity index per sampling site in Italy. *p < 0.05; ***p < 0.01.

**FIGURE 6:**
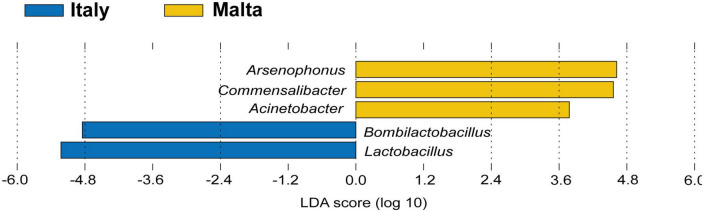
LEfSe analysis highlighting possible microbial biomarkers of the Maltese (lineage A) and Italian (lineage C) honeybees.

The gut microbiota composition of honey bees in Malta (lineage A) showed major differences when compared to the Italian honey bees (lineage C) with significant differences detected in microbial diversity within locations at genus level Figures 4A–K. Honey bees collected in Malta showed significant increases in microbial groups such as *Bartonella* (31.26% in lineage A *vs.* 4.82% in lineage C), *Bombella* (2.280% in A *vs.* 0.005% in C) and *Commensalibacter* (5.59% in A *vs.* 0.73% in C) (Figures 4B, E, F, *p* < 0.01). Notably, *Bartonella* was found to be the most highly represented genus in almost all sampled Maltese honey bees’ guts, with the sole exception of ZT2 and ZT10 which were dominated by *Arsenophonus* (89.34 and 98.77% in ZT2 and ZT10, respectively Figure 4A) and CM9, GH10, and ZT5 which were dominated by *Snodgrassella* (from 27.10 to 63.76%, Figure 4J). On the contrary, major core microbial groups *Bombilactobacillus* and *Lactobacillus* for Lactobacillaceae (Lactobacillaceae: 14.86% in lineage A *vs* 61.50% in lineage C, Figures 4D, I and Supplementary Figure 9), *Frischella* (3.60% in A *vs.* 5.92% in C, Figure 4G), and *Gilliamella* (10.17% in A *vs.* 14.12% in C, Figure 4H) were found at a significantly lower proportion in honey bees collected from Malta (*p* < 0.05). Other core microbial groups like *Bifidobacterium* (Figure 4C) and *Snodgrassella* (Figure 4J) did not significantly vary among honey bees sampled in Malta and in Italy. Figures 5A, B report the bar charts and the differentially abundant genera, comparing the composition of the Malta and Italy sampling sites. Comparison of the major microbial genera per sampling site (BZ, CM, GH, VS, SLS, ZT) are reported in Supplementary Figures 10A–K, among samples in Supplementary Figure 11 and raw data per for the major microbial taxa per sample are reported in Supplementary Table 3. Bacterial within-sample diversity of the Maltese sampling sites (lineage A, localities CM, GH, and ZT) or the Italian ones (lineage C, localities BZ, SLS, and VS) did not significantly differ for neither observed ASVs nor Shannon α-diversity indexes (CM *vs.* GH *vs.* ZT and BZ *vs.* SLS *vs.* VS). However, when the sampling sites of Italy and Malta (BZ, SLS, and VS *vs.* CM, GH, and ZT) were compared, observed ASVs and Shannon indexes resulted in significant differences (*p* < 0.01, Figures 5C, D). Additionally, the bacterial community compositions were significantly different when comparing honey bees sampled in Italy to those sampled in Malta, as evidenced by the Bray–Curtis dissimilarity index (Figures 5E–G) and the Unweighted Unifrac β-diversity metrics (Supplementary Figure 12). LEfSe analysis (Figure 6) confirmed the significant fold change of some ASVs between Malta and Italy: *Bombilactobacillus* and *Lactobacillus* are more abundant in honeybees sampled in Italy (Lineage C) whereas *Commensalibacter*, *Acinetobacter*, and *Arsenophonus* resulted with an increased abundance in Maltese honey bees (Lineage A).

Comparison of the predicted metabolic pathways of the honey bee microbiomes in Malta and Italy showed a clear separation between the two mitochondrial haplotypes (lineage A and C) (Supplementary Figure 13). In more details, Italian bees showed increased predicted abundance of genes involved in terpene biosynthesis, formaldehyde oxidation as well as lactose and galactose degradation. Maltese bees had increased predicted abundance of genes involved in tryptophan metabolism and B12 vitamin production (Supplementary Figure 14).

### Lactobacillaceae counts, grouping, and identification

Lactobacillaceae from the three sampling locations in Malta were detected in high numbers and plate count enumeration showed the following: 8.67 ± 0.03 Log cfu/g (GH), 6.67 ± 0.03 Log cfu/g (ZT) and 7.28 ± 0.02 Log cfu/g (CM) of gut content. The cluster analysis of random amplification of polymorphic DNA (RAPD) profiles of 184 isolated colonies showed a large heterogeneity, although most of the isolates belonging to the same sampling site, to some extent, clustered together. In some cases, the cluster similarity was over 90% (Supplementary Figure 15); overall, 36 lactobacilli belonging to the corresponding different clusters were processed for sequencing and the taxonomic identification is shown in Supplementary Table 4. Based on the percentage identity of the 16S rRNA gene of the isolates with the sequences in the NCBI database, the majority of Lactobacillaceae strains isolated from the modified MRS agar showed the greatest similarity to *A. kunkeei* (the nucleotide identity was over 99%). The obtained phylogenetic tree (Supplementary Figure 16) showed three main clusters of *A. kunkeei*. However, the *A. kunkeei* isolates from Malta and other countries (especially Sweden and Germany) did not group into specific clusters but mixed into the three distinct clusters.

### PCR-DGGE results

The DGGE profiles obtained from each sample had several major PCR bands and a characteristic pattern of bands was detected in each locality. The cluster analysis (Supplementary Figure 17) highlighted three major clusters (cut off at 56%). GH samples clustered together, and the similarity was over 85% for most profiles. The biggest cluster, divided in different sub-clusters, comprises all the profiles from ZT and half from CM (similarity was less than 80%). Similarity above 90% was associated with only a few profiles belonging to the same sampling site. Finally, the third cluster was related to nine profiles from CM with six of them having a very similar visual profile. The Shannon–Wiener diversity index and the Simpson index did not differ among samples and the evenness was significantly lower in GH samples when compared to CM and Z. DNA sequences of 45 bands corresponded to different Lactobacillaceae (Supplementary Figure 15).

### Results of the climate analysis

**FIGURE 7:**
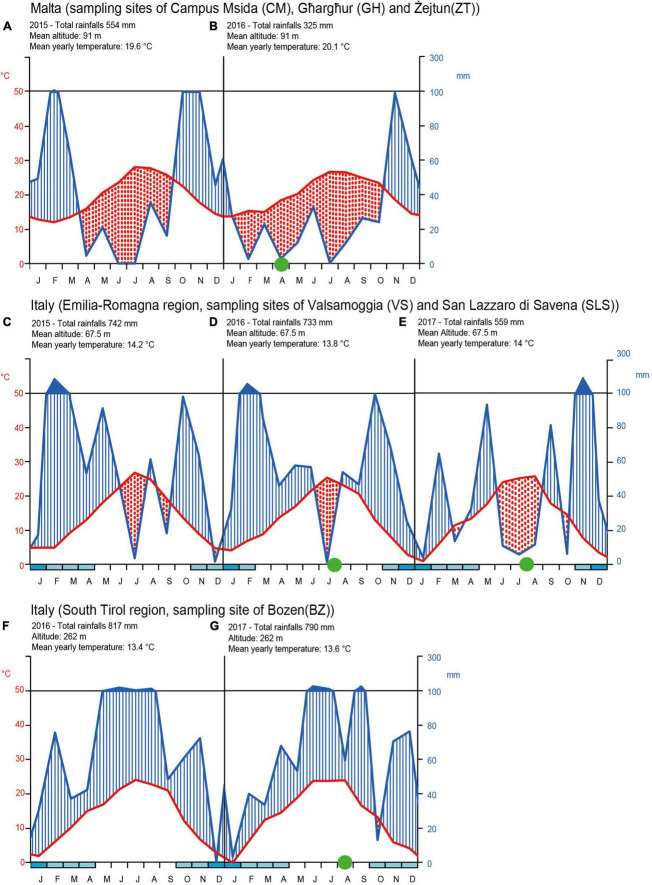
Walter and Lieth climate diagrams for drought periods in Malta (sampling sites CM, GH, ZT) in the years 2015 **(A)** and 2016 **(B)**. In Italy for the Emilia-Romagna region sampling zones of VS and SLS in the years 2015 **(C)**, 2016 **(D)**, and 2017 **(E)**. In Italy for the South Tirol region sampling zone of BZ the years 2016 **(F)** and 2017 **(G)**. The diagrams shows on the x-axis the months of the year and on the ordinate the rainfall amount (on the right) and temperatures (on the left). The temperature values are shown on a scale double that of precipitation (1°C = 2 mm). When the precipitation curve (blue line) drops below that of the temperature (red line) the period concerned is considered as drought. Finally, if monthly rainfall values exceed 100 mm, the rainy period is represented ten times smaller than that previously adopted scale for rainfall lower than 100 mm. Blue marks in the x-axis represents period of intense frost. Green circles represent the honey bee sampling period for this work and for retrieved data from Alberoni et al. (2021a,b) and Baffoni et al. (2021).

The results of the climatic analysis are shown in Figure 7. Climate data show that the island of Malta is affected by severe and long-lasting periods of drought, quantifiable to 6 months in 2015 and 9 months in 2016 (year of sampling of the Maltese honey bees). The drought period was shorter in the Emilia Romagna region of Italy, with 2 months of drought in 2015, 1 month in 2016, and 3 months in 2017 (2016 and 2017 are the years of sampling). Finally, in the Italian province of South Tyrol, no drought was detected in either 2016 or 2017.

## Discussion

In honey bees, the core gut bacterial microbiota is relatively stable, comprising five to eight bacterial taxa specialized in terms of metabolic capabilities (Maes et al., 2016; Motta et al., 2020). Variations within core bacterial taxa proportion are usually driven by environmental or rearing conditions such as seasonality (Kešnerová et al., 2020; Castelli et al., 2022), diet and feed additives (Maes et al., 2016; Alberoni et al., 2021b), xenobiotics (Motta et al., 2020) or pathogens (Alberoni et al., 2022; Jabal-Uriel et al., 2022). The proportions of the core microbial genera, or their presence/absence, directly influence the functionality of the gut microbiome, affecting honey bees’ behavior through impairment of the gut-brain axis (Zhang et al., 2022a,b) and efficiency in nutrient digestion (Alberoni et al., 2022). In addition, Powell et al. (2016) showed how lineages of gut bacteria often include many closely related strains, not distinguishable at species level but highly specialized and restricted to a single host species or subspecies. Recently, Su et al. (2022) studied the impact of both host genetics and diet on the gut microbial populations of different *Apis ceranae* subspecies. The results showed extensive overlapping of the gut microbial strains among different subspecies and suggested an effect of the floral diet in maintaining specialized bacterial traits.

The relationship between microbial population and the environment is therefore a new frontier in the understanding of the honey bees’ microbiome’s structure and functionality. In this study we tried to contribute to this understanding by focusing on a unique Mediterranean habitat, the Maltese Islands, characterized by (i) a semi-desert climate with intense drought periods, (ii) the presence of Mediterranean plants producing nectars with high amount of essential oils (*e.g., Thymus*), (iii) the proximity to the sea of the entire territory with the impact of salinity and high humidity, and (iv) the isolation of the honey bee ecosystem characterized by an African lineage population resistant to *Varroa destructor*.

These contexts are probably the reasons why this study has identified marked differences in the core gut microbial community of Maltese honey bees (lineage A) when compared to the Italian honey bees (lineage C). Even though all the eight core microbial taxa were present in both the Maltese and Italian honey bees, the proportions were different. The Maltese honey bees showed an inverse proportion of Lactobacillaceae and Bartonellaceae when compared to Italian samples. In European honey bees (both C and M lineages), *Lactobacillus* and *Bombilactobacillus* altogether are much more represented, whereas *Bartonella*, although still considered a core member, is only present as a minor group. In the Maltese honey bees (lineage A), these proportions are inverted to such an extent that *Lactobacillus* and *Bombilactobacillus* are highlighted as biomarkers of lineage C honeybees in the LEfSe analysis. The same concept can be applied to *Bartonella* and Acetobacteraceae (*Commensalibacter* and *Acinetobacter*) for the Maltese honey bees. However, to consider some taxa as biomarkers, a further validation with additional analyses is envisaged. Recent publication focusing on the gut microbiota of another lineage A honey bee, *Apis mellifera scutellata* (Kenya), did not highlight a similarly predominant population of *Bartonella* (Tola et al., 2020). As such, we suggest that the preponderance of *Bartonella* in *Apis mellifera ruttneri* is related to the Maltese environmental conditions rather than the lineage itself, even if additional factors such as host genetics, seasonality, or geography, in synergy with each other or interacting with environmental factors, may still be plausible. Regarding the environmental conditions that may play a major role in the microbiome acquisition, the influence of nectar and pollen composition and climatic conditions are hypothesized as driving factors in the shaping of the core microbiota. It is known that environmental conditions characterized by high solar irradiance, high temperature and humidity can strongly increase the polyphenolic content of plant tissues (Spayd et al., 2002) and, consequently, also the polyphenolic content in honey (Tenore et al., 2012). *Bartonella apis* was found to harbor genes for the degradation of secondary plant metabolites, such as 4-hydroxybenzoate and quinate (Segers et al., 2017), but also hydrocarbons in crude oil (Bacosa et al., 2015) and organophosphorus insecticides like fenitrothion (Tago et al., 2006). It can therefore be postulated that *Bartonella* can degrade a large array of aromatic compounds and terpenes, leading to a positive selection in the Maltese honey bees as adaptation to nectars with higher content of phenolic compounds (Mannina et al., 2015). *Bartonella*, therefore, provides crucial functions for its host and might be considered a typical trait of the Maltese honeybees. Further studies are envisaged to isolate and characterize *Bartonella* strains from this source. Another factor that might have led to an increased abundance of *Bartonella* is the scarcity of available nectar. During the sampling season, the Maltese Island was in a condition of severe drought with scarcity of nectar. Kešnerová et al. (2020) highlighted that *Bartonella* population increases in wintering bees in Switzerland, that is during a period of absence of nectar. Although a detailed metabolic analysis of the single detected taxa has not been performed in this work, a separation of the predicted metabolic functionality of the Italian and Maltese honeybee gut bacteria has been observed and appears to be related to the unique Maltese habitat.

While *Bartonella*, *Bombella* and *Commensalibacter* in Maltese honey bees were observed with high abundance, *Bombilactobacillus*, *Frischella*, *Gilliamella*, and *Lactobacillus* were low in abundance. Our results report not only a low abundance of total Lactobacillaceae, but also a significant change within the Lactobacillaceae genera. *Bombilactobacillus* population in Maltese honey bees was very low when compared to the Italian honey bees. Previous works have correlated this reduction to antibiotic treatments or xenobiotic stressors (Raymann et al., 2017; Motta et al., 2018, 2020; Alberoni et al., 2021a,b; Baffoni et al., 2021). Also, *Lactobacillus* abundance was significantly lower in Maltese honey bees in comparison to the Italian honey bees analyzed, whereas *Apilactobacillus*, whose members are typical colonizers of the honey bee’s honey stomach (not analyzed in this work), was found abundant in the Maltese honey bee midgut and rectum. To the best of our knowledge, the high abundance of *Apilactobacillus* is atypical in any analyzed western honey bees. NGS results were also confirmed by plate isolation in MRS medium of Lactobacillaceae, where most isolated strains belonged to *A. kunkeei*. Moreover, DGGE analysis showed a noteworthy strain variability within *A. kunkeei* despite the low abundance in the gut microbiome. Strain variability within the same microbial taxon in samples of different geographical locations was also highlighted by Moran et al. (2012), Anderson et al. (2013), and Engel et al. (2014).

*Commensalibacter*, *Bombella* and *Pseudomonas* were found in higher abundance in the Maltese honey bees when compared to the Italian honey bees. *Bombella* and *Pseudomonas* are usually occasional colonizers of the honey bee gut in European honey bees. *Commensalibacter* is a controversial non-obligatory core member of the honey bee microbiota or even classified as core hive microorganisms rather than core gut microorganism of adult bees (Corby-Harris et al., 2014). The definition of core microbiome considers different variables such as frequency and abundance (Ainsworth et al., 2015; Risely, 2020). In the case of honey bees, *Bifidobacterium*, the prevalent genus within Actinobacteria in *A. mellifera ligustica* gut using a culture-independent analysis (Cui et al., 2022a), is classified as a core microbial taxon despite its low relative abundance (usually around 2% reaching 5% in some cases) because of its prevalence. Therefore, the separation between core and non-core taxa remains challenging in insects. Our results suggest that *Pseudomonas* still shows a low prevalence within the gut microbiome of the Maltese honey bee and cannot be considered as a core taxon even if its relative abundance in some samples is high. On the contrary, *Bombella* and *Commensalibacter* showed a relative abundance similar to *Bifidobacterium* in most of the samples, therefore they might be considered as core members of the Maltese honey bee. These results also find a confirmation in *Apis mellifera scutellata* in which *Bombella* and *Commensalibacter* are also described as core microbiome taxa (Tola et al., 2020). Higher occurrence of *A. kunkeei* and *Bombella* has been correlated with diet change (presence, absence, or degraded pollen) and stress (Anderson and Ricigliano, 2017) and recently it has been shown to be negatively correlated with yeasts abundance in the honey bee ileum and rectum (Anderson et al., 2022). This highlights the possible influence the Maltese climate and environment has on the local honey bees’ gut microbial population. *Bifidobacterium* did not significantly vary among the different honey bees subspecies and its relative abundance was in overall agreement with Cui et al. (2022a).

*Arsenophonus* is a horizontally transmitted symbiont in honey bees (Drew et al., 2021) that, in this study, was detected only in four Maltese honey bee samples although with relevant abundance. *Arsenophonus* can be an insect reproduction manipulating parasite (Elston et al., 2022) that can potentially colonize off-target microbial niches; therefore, it should be intended as a non-core gut bacterial community member. Little is known about this genus, however, recently, a novel species, *Arsenophonus apicola*, was isolated and characterized in honey bees (Nadal-Jimenez et al., 2022). Its abundance is linked with seasonality, increasing in honey bees during winter while almost disappearing in the spring (Drew et al., 2021). *Arsenophonus* also correlates with areas of anthropogenic pressure and intensive agriculture (Gorrochategui-Ortega et al., 2022), which are reflective of the Maltese Islands. In many insects, *Arsenophonus* is a harmful intracellular parasite, for instance negatively influencing reproduction in *Nasonia* wasp (Darby et al., 2010). There is little evidence supporting the pathogenicity of *Arsenophonus* in honey bees, although analyses of the gut microbiome of honey bees with colony collapse disorder symptomatology showed an increase of this taxon (Cornman et al., 2012). Also, Budge et al. (2016) associated *Arsenophonus* with poor honey bee health due to high viral load, however, this does not prove its pathogenicity. Yet its presence was found in *V. destructor*, a possible vector of infection for honey bees (Hubert et al., 2015).

The Walter and Lieth climatic analysis confirmed a persistent and very dry climatic conditions on the Maltese island, which, also based on historical data, has determined the selection of a spontaneous Mediterranean flora. Although the Emilia-Romagna region undergoes periods of drought, these are shorter and consequently, the spontaneous flora differs in the two areas (Galuzzo et al., 2021). In the Emilia-Romagna region, the spontaneous vegetation is continental (large latifolia plants) and in the two sampling areas, not of the Mediterranean type. Crops and fruit trees are also very different in the two areas. The Emilia-Romagna region spontaneous flora resembles more the alpine vegetation rather than the Mediterranean one and this may explain the results on the bacterial community analysis of honeybees sampled in Italy, which all cluster close, highlighting a well-defined and stable core microbiota despite differences in climatic and environmental conditions of the two sampling areas (Emilia-Romagna and the South Tyrol regions). Therefore, sampling sites that are hundreds of kilometers in distance and with different prevalent honey bees subspecies (South Tyrol = *A. mellifera carnica*; Emilia-Romagna = *A. mellifera ligustica*), show remarkable stability of the core microbial groups between sites and over time. On the other hand, the intestinal microbial communities of the Maltese bees (lineage A) showed a dispersed spatial distribution. The microbiota seemed less consistent in the abundance of core microbial taxa although differences among sites were not significant and it harbored a relevant number of low-abundant microbial genera (below 1%), similar to honey bees treated with antibiotics (Baffoni et al., 2021) and suffering gut dysbiosis.

Finally, the yeast community found in the Maltese honey bees showed an important presence of Metschnikowiaceae members, mainly represented by the genera *Metschnikowia* and *Kodamaea*. Little is known about the effect of yeasts on honey bee health, but recent studies have shown that yeasts, when supplied as additives to the honey bee diet, may have an immunomodulatory function controlling the transcription of immune-related genes and they can also alter the bacterial composition of the gut with unpredictable effects (Tauber et al., 2019). Although studies in the literature are not conclusive on this point, it has been highlighted that yeasts are likely associated with both negative and positive aspects of every stage of the honey bee’s life that needs to be further explored (Ptaszyńska et al., 2016; Tauber et al., 2019). Anderson et al., 2022 suggested that fungi or fungal associated factors contribute to core-hindgut microbiota assembly especially in the ileum, however, the abundance and prevalence of *Bombella* and *A. kunkeei* found in this work suggest a sparse yeast population at the sampling time of Maltese honey bees. The antagonisms of yeasts and Lactobacillace is already well known in nectar (Álvarez-Pérez et al., 2019) and may also occur in the gut microbiome. *Metschnikowia* genus is reported as a nectar-specialist yeast that, living in the flower nectar, plays an important role in honey bees’ attraction and thus in flower and crop pollination (Good et al., 2014; Colda et al., 2021). When consumed by pollinators, the nectar microorganisms, in particular yeasts, may serve as an additional source of nutrition (*e.g.*, vitamins and steroids), that may have positive effects on the flower visiting insects (Martin et al., 2022), although this mechanism has been poorly studied. *Metschnikowia* species, although different from those identified in this work, have been isolated from the honey bee gut (Good et al., 2014). However, no isolation of the species detected in our work has been documented so far. A recent work by Cui et al. (2022b) explored the phylogenetic diversity and community composition of *A. mellifera ligustica* associated fungi in honey bees and the colony environment, including the gut and bee-derived products using a combination of culture-dependent and culture-independent approaches. The relative abundances of ASVs showed data similar to ours at the phylum level, with a highest abundance of Ascomycota followed by a lower proportion of Basidiomycota. Data at genus level (Cui et al., 2022b) showed a relative abundance of *Kodamaea* higher than 80%, different from our results that recorded this genus at 8%, on the other hand, *Metschnikowia* was not detected at all (threshold 0.1%). The *Metschnikowia* genus was, on the contrary, detected at 18% of relative abundance in honeycomb in the same study. Our study has considered a different bee subspecies and it is difficult to extrapolate conclusions considering the small amount of data present in the literature on the yeast gut population. Our study highlights the need to further explore the impact of yeasts in honey bee physiology and gut microbial population.

In conclusion, the Maltese honey bee was found to host a peculiar core microbiome, where *Apilactobacillus*, *Bartonella*, *Commensalibacter*, and *Bombella* were among the major taxa at the expense of *Frischella*, *Gilliamella*, and *Lactobacillus*. With currently available data on gut microbes in Maltese honey bees, obtained over a single sampling time point, it cannot be clearly assumed that the peculiar gut microbial composition of the Maltese honey bee is ascribed to the different evolutionary phylogenesis of this subspecies. Multiple samplings along the season are needed to separate the contributions of honey bee genetics and environmental influence. The environment seems the major driving factor shaping the local flora, food availability and therefore the honeybee microbial population although other co-occurring factors cannot be excluded. In particular, the combination of environment and genetic evolution already shown in plants (Oyserman et al., 2021) is the most likely also in honeybees, although further studies are necessary to understand this combined effect. This work opens to future research that focuses on the ability of different honey bee subspecies to select and co-evolve with specific microbial taxa and strains, adapting to the local environment. This work also evidences the importance of research on honey bees’ microbiome adaptation to climate conditions (especially drought), in a world facing strong climate changes.

## Data availability statement

The datasets presented in this study can be found in online repositories. The names of the repository/repositories and accession number(s) can be found in the article/Supplementary material.

## Author contributions

DA, FG, and DM collected honey bees samples. DA performed DNA extraction. DA, FG, and LB prepared NGS sequencing on bacteria. RJ and FG prepared NGS sequencing on yeasts. FG carried out DGGE analysis and the isolation of microbial strains. LB and RJ performed bioinformatics analysis. RJ, DA, and LB were involved in statistics and figures plotting. FG, DA, RJ, SC, and DD were involved in the manuscript writing. DM, DD, and DN were involved in the research funding. All authors contributed to the article and approved the submitted version.

## Footnotes

1
www.ncbi.nlm.nih.gov/genbank/

2
http://www.ncbi.nlm.nih.gov/BLAST/

3
https://weatherspark.com/y/148306/Average-Weather-at-Malta-International-Airport-Malta-Year-Round

4
https://data.civis.bz.it/it/dataset/misure-meteo-e-idrografiche

5
https://simc.arpae.it/dext3r/
