# Supplementary material for: Environment or genetic isolation? An atypical intestinal microbiota in the Maltese honey bee Apis mellifera spp. ruttneri
Source: Front Microbiol. 2023 Feb 23;14:1127717. doi: 10.3389/fmicb.2023.1127717 (PMC9995969; doi:10.3389/fmicb.2023.1127717)
Supplement: Supplementary file 1 [file Data_Sheet_1.pdf]

# *Supplementary Material*

**Environment or genetic isolation? An atypical intestinal microbiota in the Maltese honey bee *Apis mellifera* subsp. *ruttneri***

**Author: Francesca Gaggìa[1]†, Rasmus Riemer Jakobsen[2]†, Daniele Alberoni[1]*, Loredana Baffoni[1], Simone Cutajar[3], David Mifsud[3], Dennis Sandris Nielsen[2], Diana Di Gioia[1]**

[1] Department of Agricultural and Food Sciences, University of Bologna, Viale Fanin 44, Bologna, 40127, Italy.

[2]Section of Microbiology and Fermentation, Department of Food Science, Faculty of Science, University of Copenhagen, DK-1958 Copenhagen, Denmark

[3]Institute of Earth Systems, L-Università tà Malta, University, Ring Rd, Msida, MSD2080, Malta.

*** Correspondence:**

Corresponding Author: daniele.alberoni@unibo.it

†These authors contributed equally to this work and share first authorship

# 1 Supplementary Figures and Tables

## 1.1 Supplementary Figures

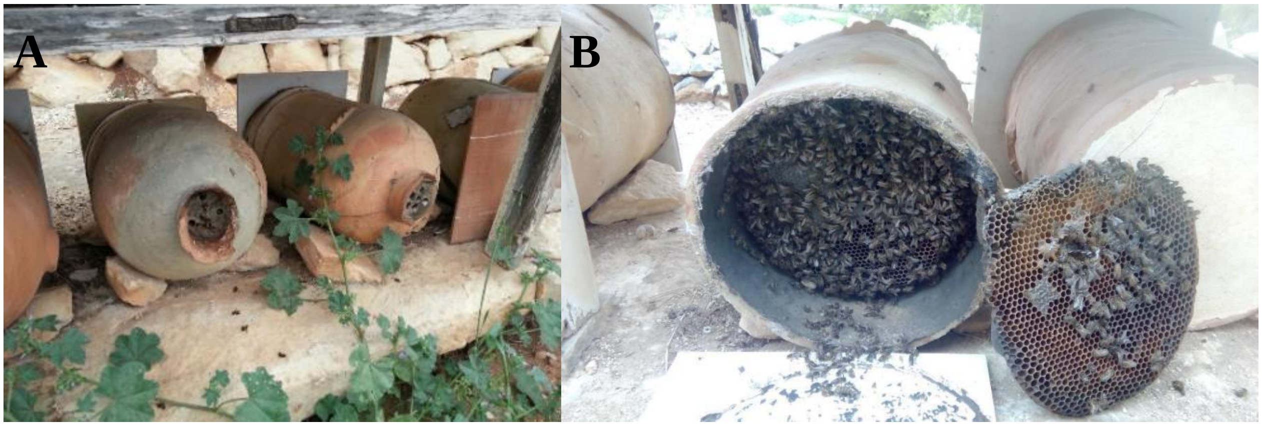


**Supplementary Figure 1A-1B**. Up until the 1950's, terracotta hives populated with *Apis mellifera ruttneri* were typical of the Maltese Island. This apiary belongs to Mario Sant, a beekeeper promoting Maltese honey bee conservation.

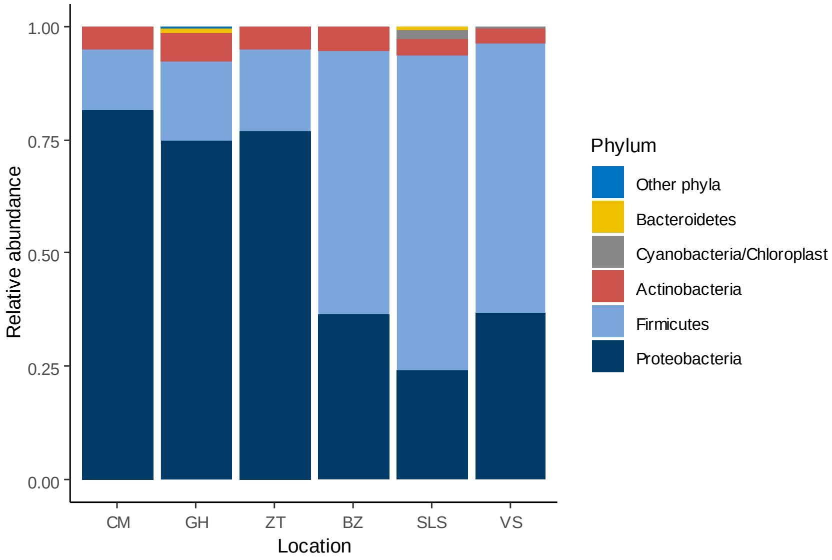


**Supplementary Figure 2.** Bar chart showing the relative abundance of bacterial phyla in the sampling sites in Malta and Italy: [CM] University of Malta – Campus Msida, [GH] Għargħur, [ZT] Żeitun, [BZ] Bozen, [SLS] San Lazzaro di Savena, [VS] Valsamoggia.

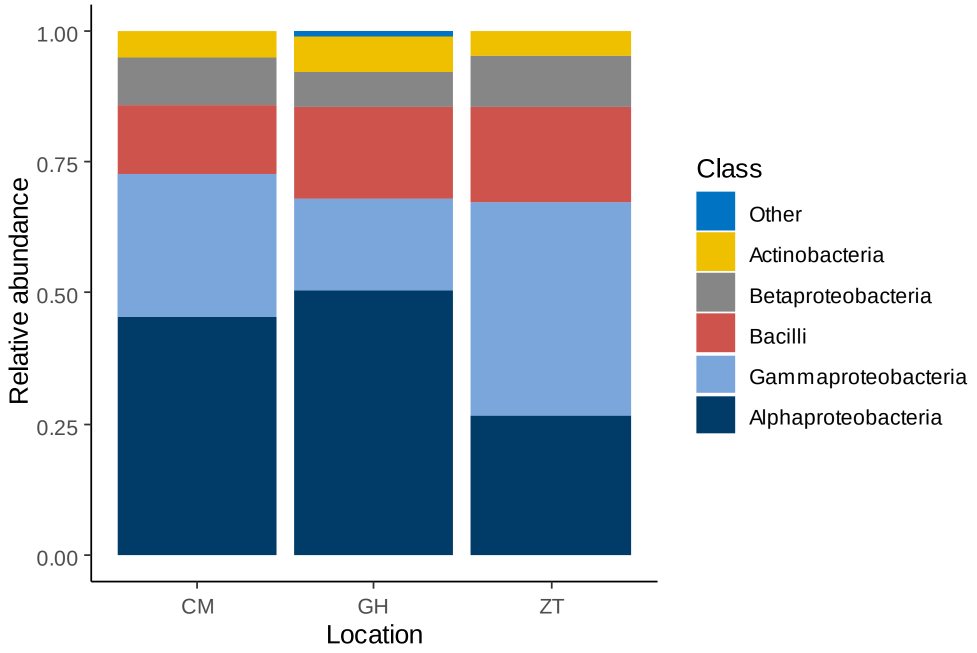


**Supplementary Figure 3.** Bar chart showing the relative abundance of bacterial classes in the sampling sites in Malta and Italy: [CM] University of Malta – Campus Msida, [GH] Għargħur, [ZT] Żeitun, [BZ] Bozen, [SLS] San Lazzaro di Savena, [VS] Valsamoggia.

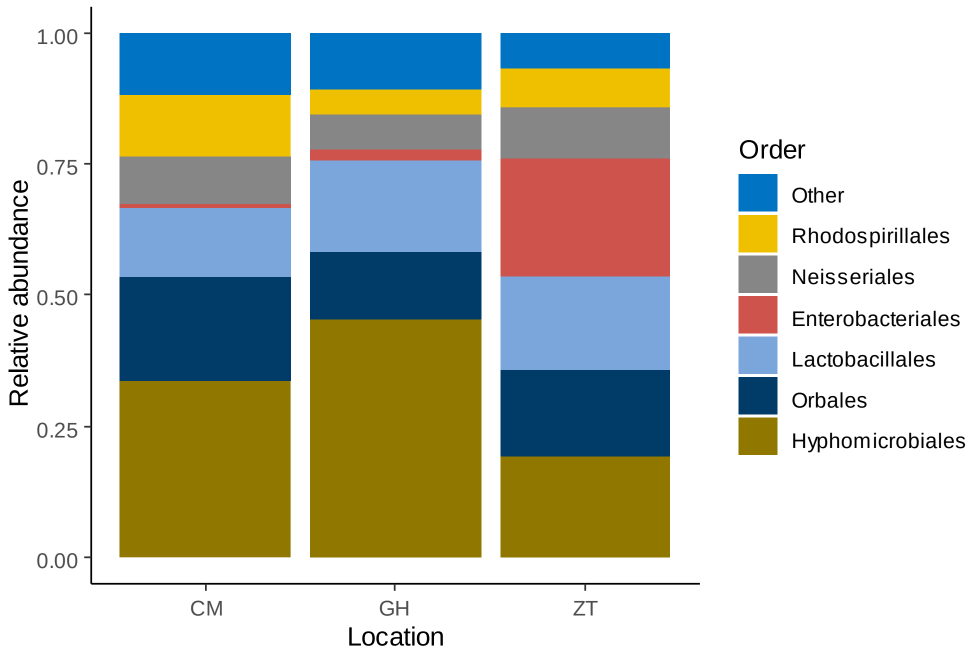


**Supplementary Figure 4.** Bar chart showing the relative abundance of bacterial orders in the sampling sites in Malta and Italy: [CM] University of Malta – Campus Msida, [GH] Għargħur, [ZT] Żeitun, [BZ] Bozen, [SLS] San Lazzaro di Savena, [VS] Valsamoggia.

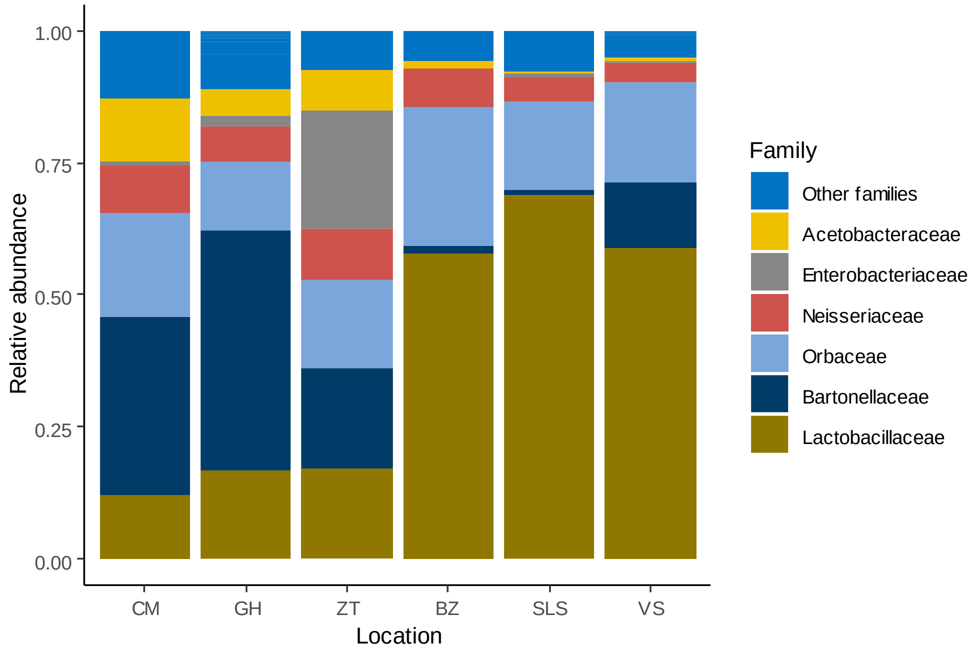


**Supplementary Figure 5.** Bar chart showing the relative abundance of bacterial orders in the sampling sites in Malta and Italy: [CM] University of Malta – Campus Msida, [GH] Għargħur, [ZT] Żeitun, [BZ] Bozen, [SLS] San Lazzaro di Savena, [VS] Valsamoggia.

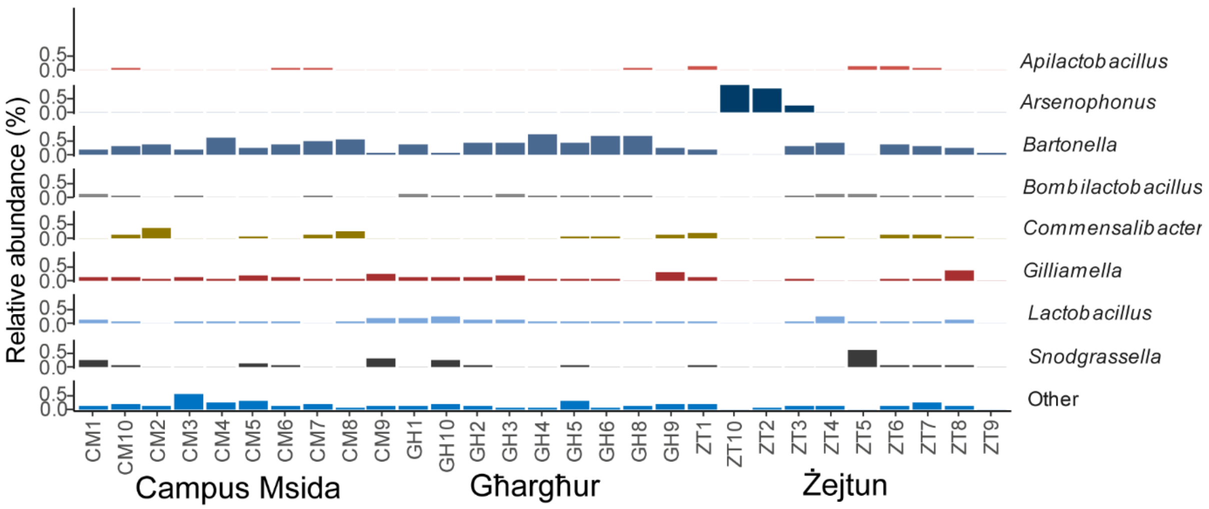


**Supplementary Figure 6**. Bar chart showing the relative abundance of the major genera of bacteria in every sampled honey bee gut in Malta.

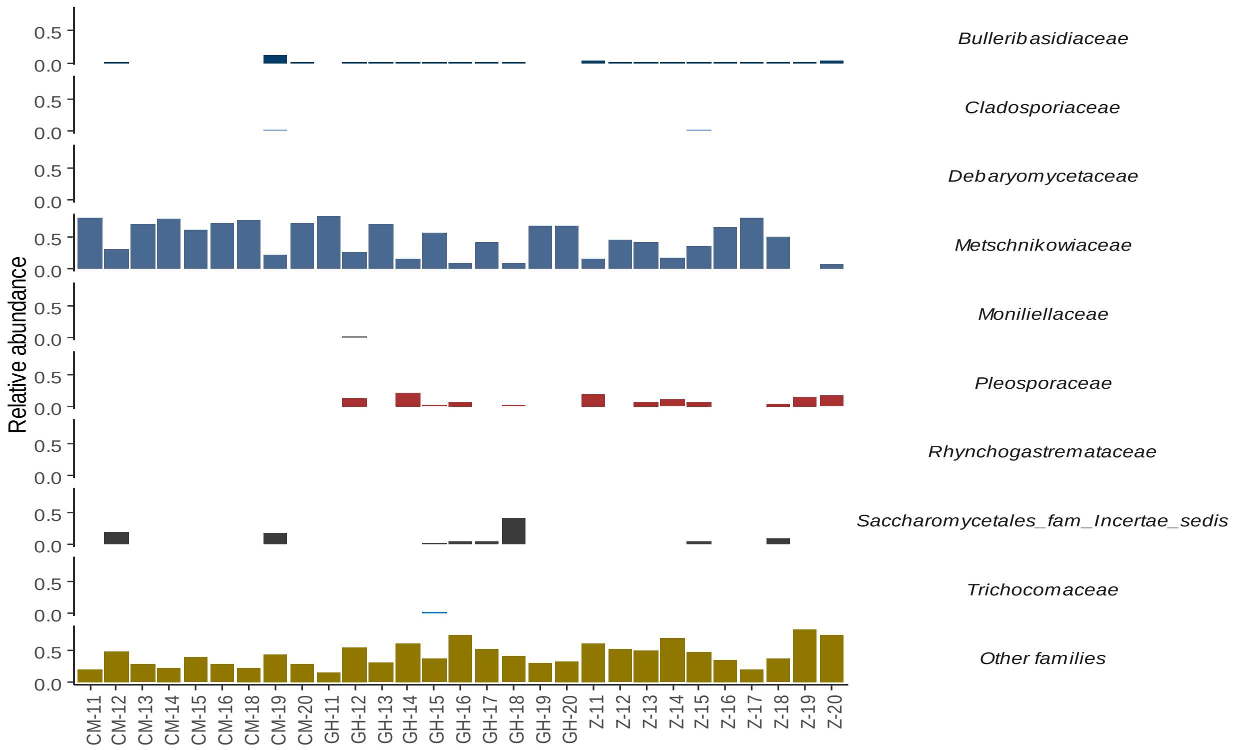


**Supplementary Figure 7**. Bar chart showing the relative abundance of the major families of fungi in every sampled honey bee gut in Malta.

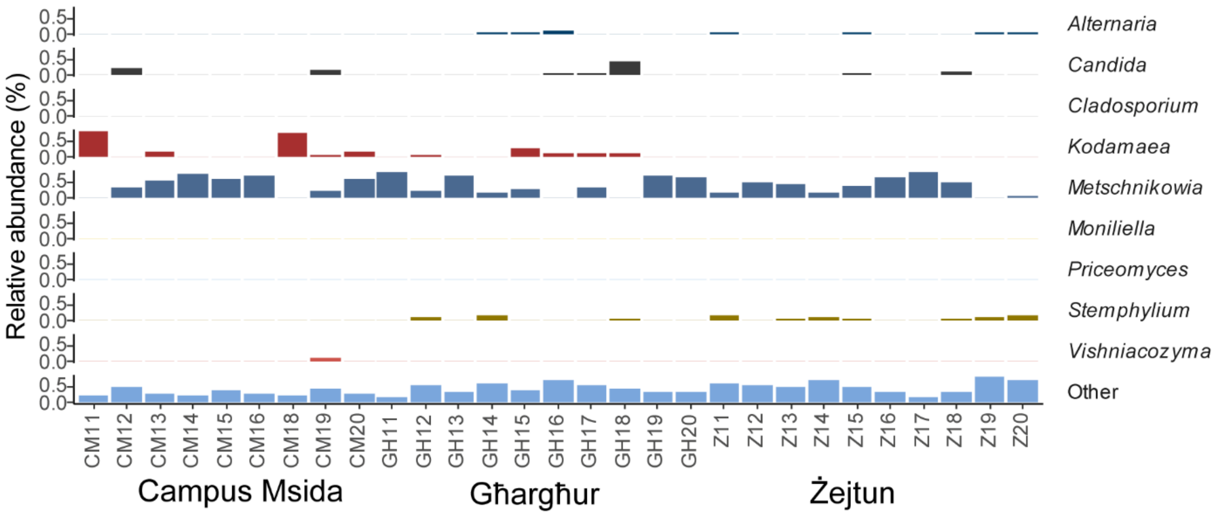


**Supplementary Figure 8**. Bar chart showing the relative abundance of the major genera of fungi in every sampled honey bee gut in Malta.

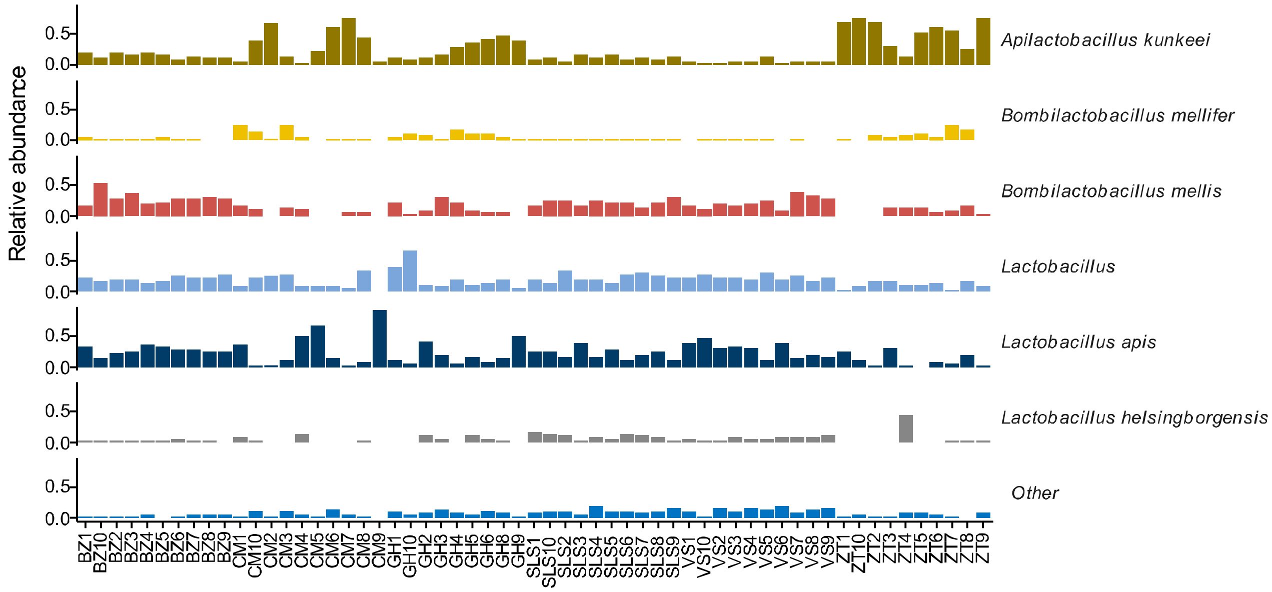


**Supplementary Figure 9**. Bar chart showing the relative abundance of the major species belonging to the family Lactobacillaceae in every sampled honey bee gut in Malta and Italy.

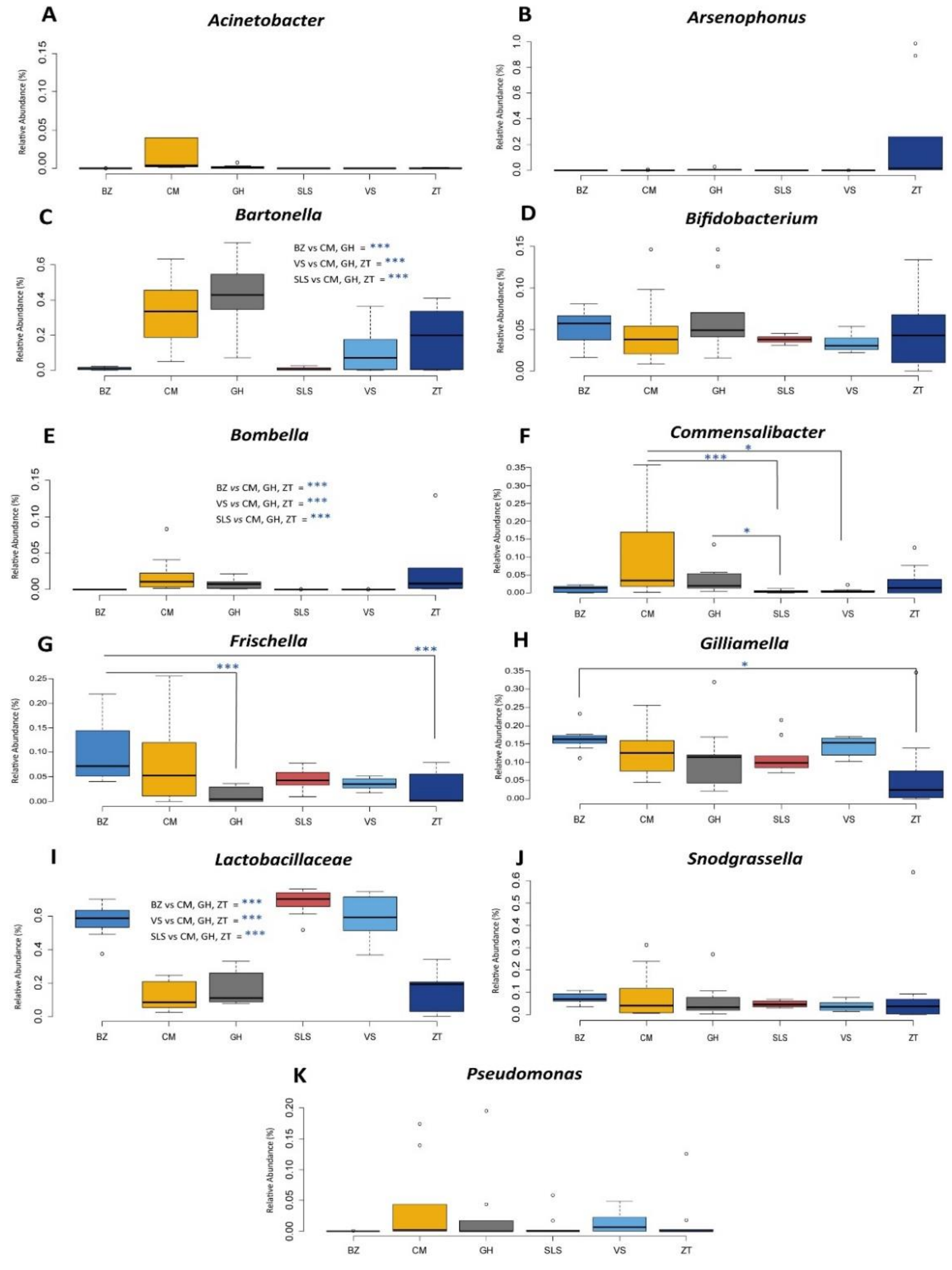


**Supplementary Figure 10A – 10K.** The boxplot charts show the relative abundance of the major microbial taxa populating the sampled honey bee guts: A) *Acinetobacter*; B) *Arsenophonus*; C) *Bartonella*; D) *Bifidobacterium*; E) *Bombella*; F) *Commensalibacter*; G) *Frischella*; H) *Gilliamella*; I) Lactobacillaceae; J) *Snodgrassella* and K) *Pseudomonas* in the different sampling sites. The sampling sites for Italy are [BZ] Bozen, South Tyrol region; [SLS] San Lazzaro di Savena, Emilia Romagna region; [VS] Valsamoggia, Emilia Romagna region, all belonging to C lineage. Sampling sites for Malta are [CM] University of Malta – Campus Msida; [GH] Għargħur; [ZT] Żeitun, all belonging to A lineage. Every sampling site account for 10 sampled honey bees. Asterisks indicate statistically significant differences (*p<0.05; ***p<0.01).

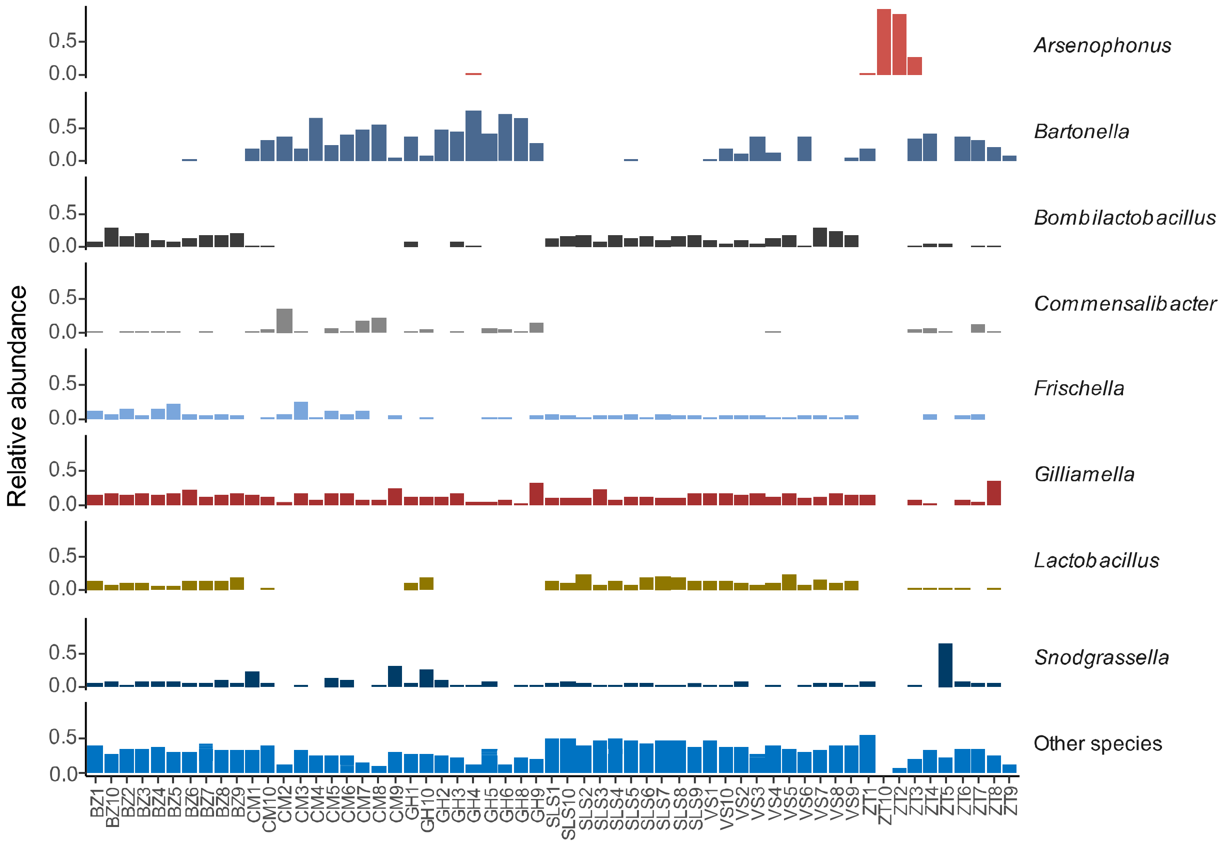


**Supplementary Figure 11.** Bar chart showing the relative abundance (%) of the major bacterial genera in every sampled honey bee gut in Malta and Italy.

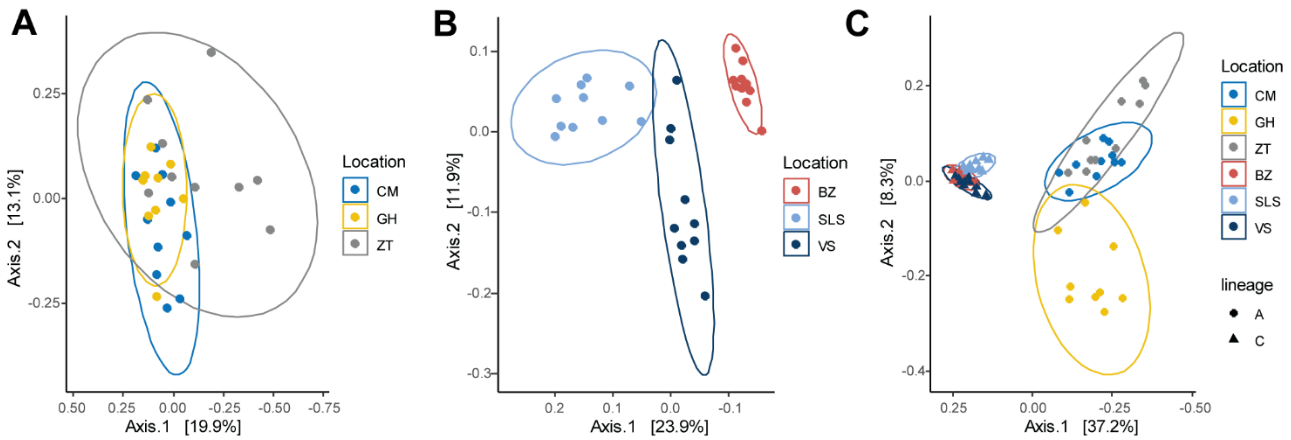


**Supplementary Figure 12. A)** β-diversity Bray–Curtis dissimilarity index per sampling site in Malta; **B)** β-diversity Bray–Curtis dissimilarity index per sampling site in Italy; **C)** β-diversity Bray–Curtis dissimilarity index per mitochondrial haplotype (lineage) and sampling site.

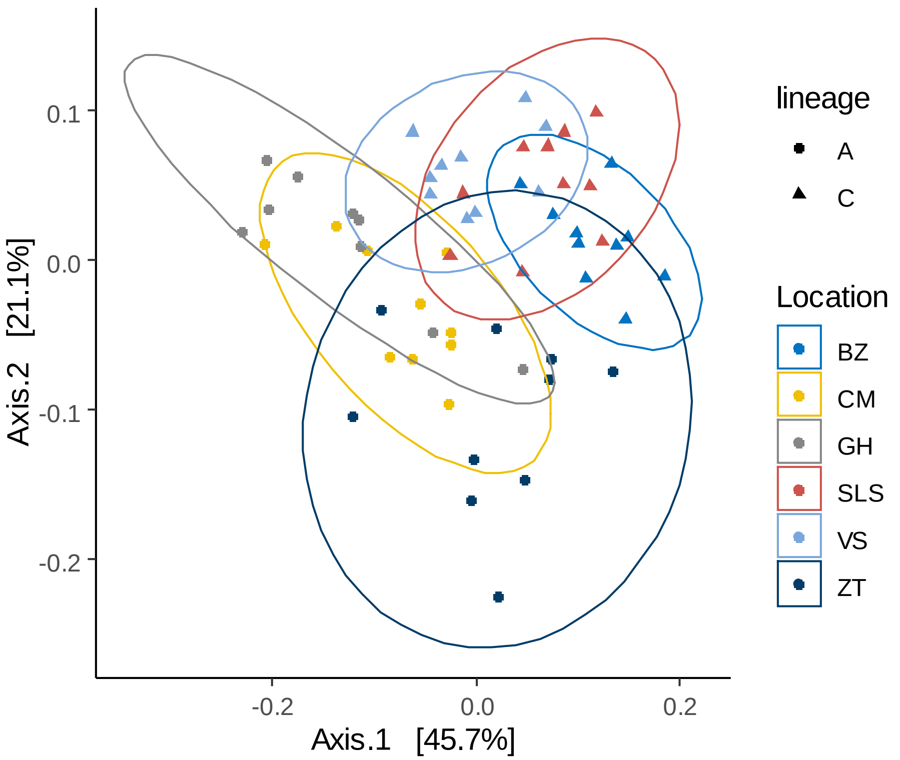


**Supplementary Figure 13.** PCA for PCRUST2 overall prediction of functionality, considering all the honey bees sampled both in Italy and in Malta, separates the lineage C honey bees from those of lineage A.

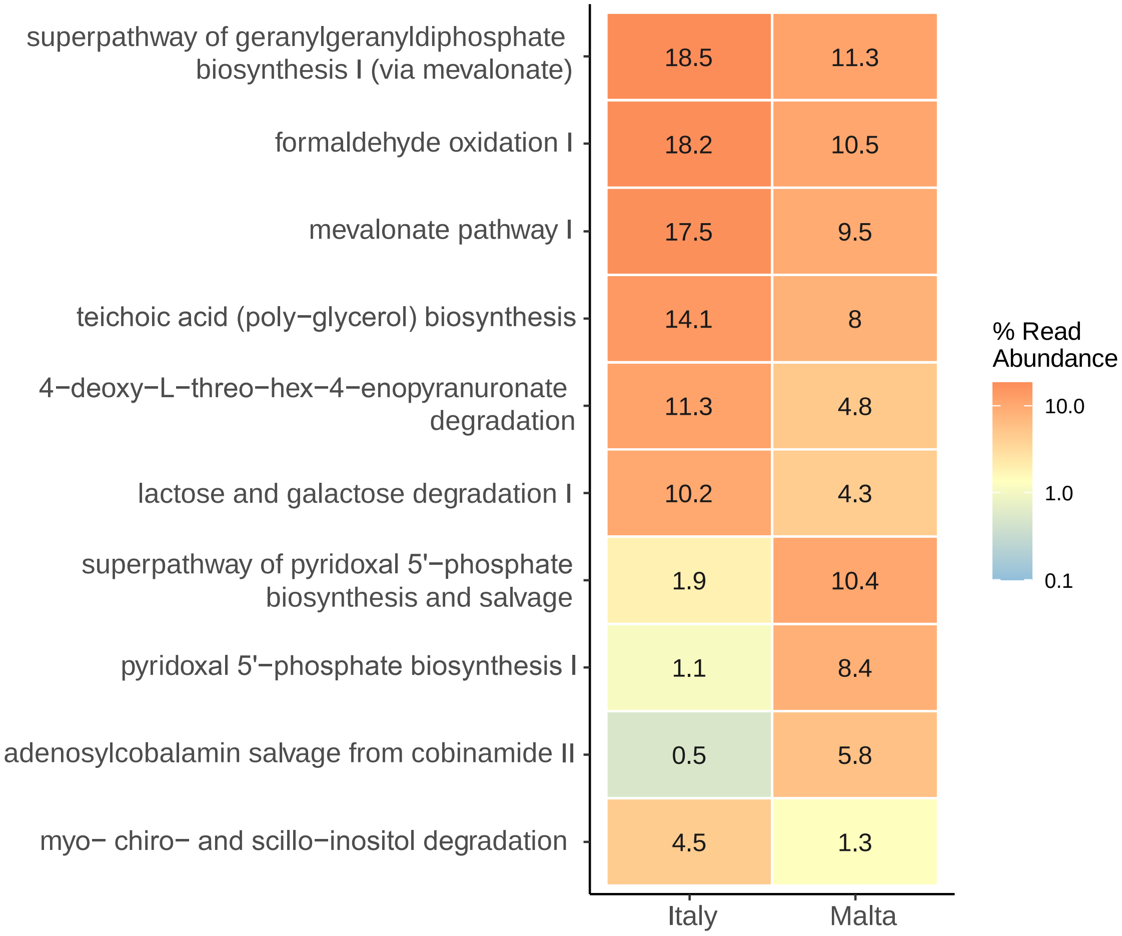


**Supplementary Figure 14.** Differential abundance heatmap highlighting significantly differentially abundant ($p<0.05$) microbial genera between honey bees sampled in Italy (lineage C) and in Malta (lineage A), showing the relative abundance of the genus;

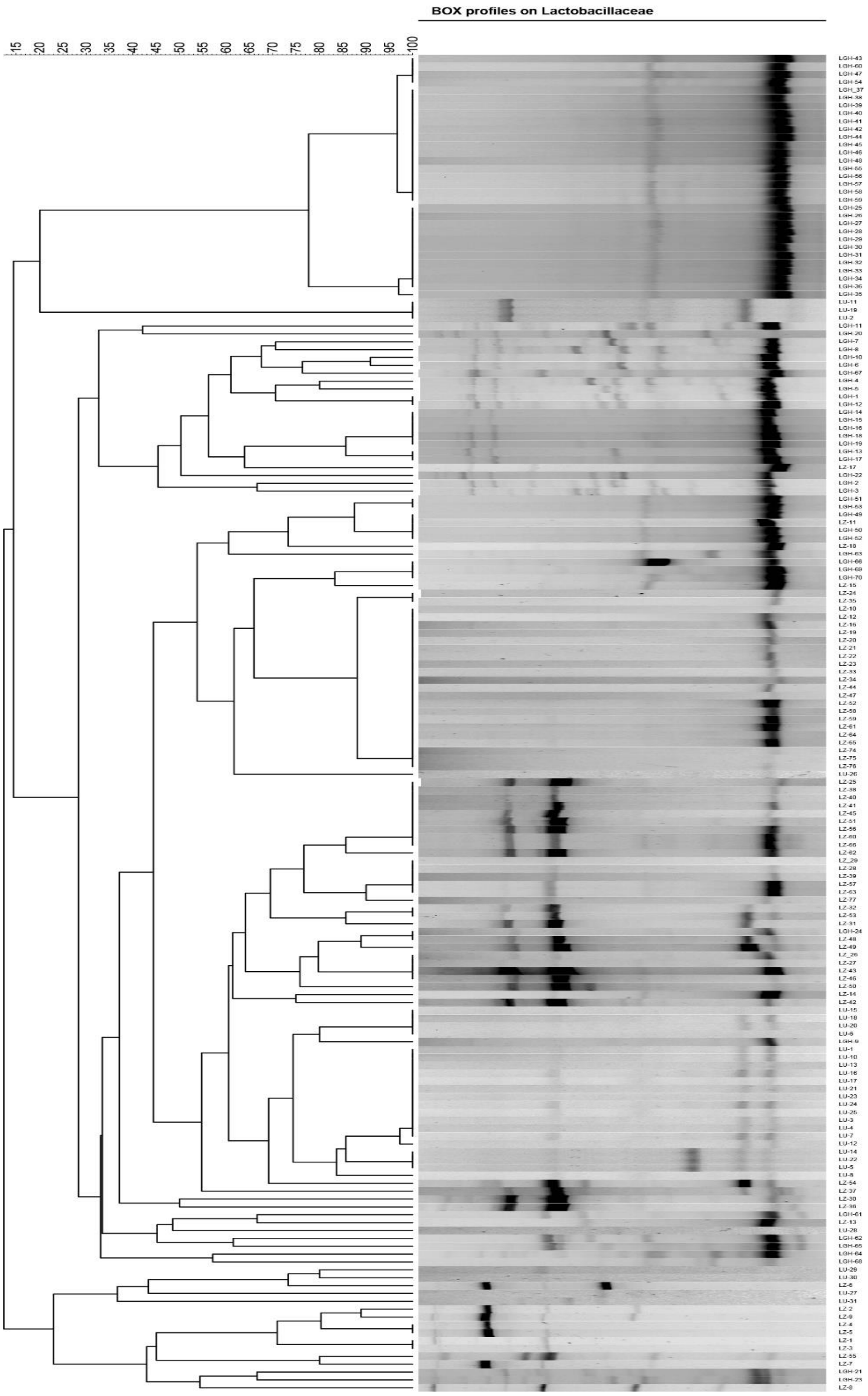


**Supplementary Figure 15.** Fingerprinting profiles on isolated Lactobacillaceae populating the gut of Maltese honey bees.

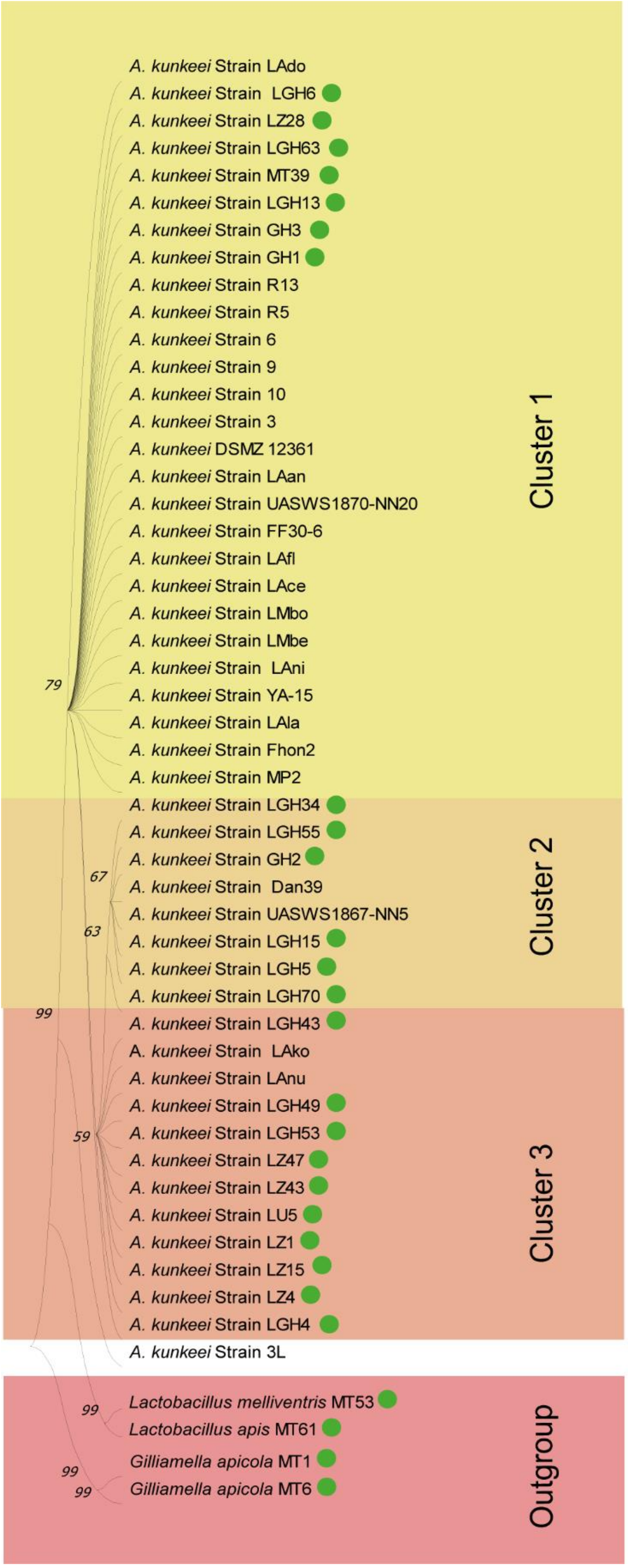


**Supplementary Figure 16.** Phylogenetic tree showing the isolated *A. kunkeei* isolated within this work (Indicated with the green dot) and other *A. kunkeei* strains retrieved from NCBI Genome RefSeq.

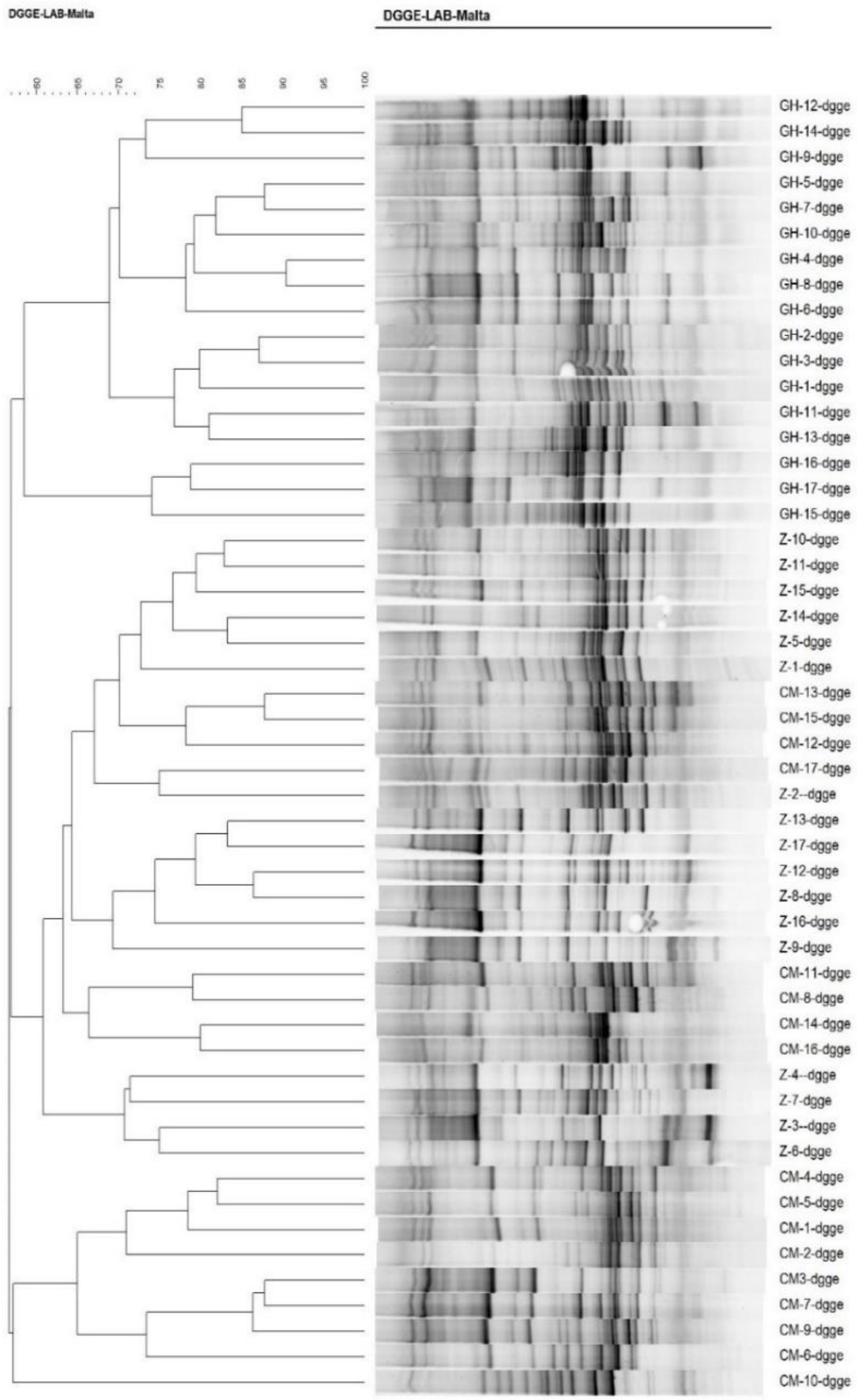


**Supplementary Figure 17.** DGGE profiles on Lactobacillaceae populating the gut of Maltese honey bees.

## 1.2 Supplementary Tables

**Supplementary Table 1.** The table reports the list of samples derived from Alberoni et al., 2021a; Alberoni et al., 2021b; Baffoni et al., 2021 research works, the original samples name and the NCBI accession number and bio-project.

| Samples name in this work | Sample name in the original works | NCBI accession | Reference |
|---|---|---|---|
| BOLZANO/BOZEN (*APIS MELLIFERA CARNICA*) | | | |
| **BZ1** | 0.CTR.1 | SAMN15728541 | Alberoni et al., 2021a (NCBI bioproject PRJNA655184) |
| **BZ2** | 0.CTR.2 | SAMN15728542 | |
| **BZ3** | 0.CTR.3 | SAMN15728543 | |
| **BZ4** | 0.CTR.4 | SAMN15728544 | |
| **BZ5** | 0.CTR.5 | SAMN15728545 | |
| **BZ6** | 0.IMID.1 | SAMN15728552 | |
| **BZ7** | 0.IMID.2 | SAMN15728553 | |
| **BZ8** | 0.IMID.3 | SAMN15728554 | |
| **BZ9** | 0.IMID.4 | SAMN15728555 | |
| **BZ10** | 0.IMID.5 | SAMN15728556 | |
| VALSAMOGIA (*APIS MELLIFERA LIGUSTICA*) | | | |
| **VS1** | T0.C1 | SAMN16442367 | Alberoni et al., 2021b (NCBI bioproject PRJNA669646) |
| **VS2** | T0.C2 | SAMN16442368 | |
| **VS3** | T0.C3 | SAMN16442369 | |
| **VS4** | T0.C4 | SAMN16442370 | |
| **VS5** | T0.C5 | SAMN16442371 | |
| **VS6** | T0.P1 | SAMN16442385 | |
| **VS7** | T0.P2 | SAMN16442386 | |
| **VS8** | T0.P3 | SAMN16442387 | |
| **VS9** | T0.P4 | SAMN16442388 | |
| **VS10** | T0.P5 | SAMN16442389 | |
| SAN LAZZARO DI SAVENA (*APIS MELLIFERA LIGUSTICA*) | | | |
| **SLS1** | T0.CA1 | SAMN16442373 | Baffoni et al., 2021 (NCBI bioproject PRJNA669646) |
| **SLS2** | T0.CA2 | SAMN16442374 | |
| **SLS3** | T0.CA3 | SAMN16442375 | |
| **SLS4** | T0.TN1 | SAMN16442409 | |
| **SLS5** | T0.TN2 | SAMN16442410 | |
| **SLS6** | T0.TN3 | SAMN16442411 | |
| **SLS7** | T4.CA1 | SAMN16442428 | |
| **SLS8** | T4.CA2 | SAMN16442429 | |
| **SLS9** | T4.CA3 | SAMN16442430 | |
| **SLS10** | T4.CA4 | SAMN16442431 | |

**Supplementary Table S2.** Retrieved *Apilactobacillus kunkeei* 16S rRNA gene sequences from the NCBI RefSeq database.

| **Strain** | **Origin** | **RefSeq** |
|---|---|---|
| *Apilactobacillus kunkeei* Fhon2 | Sweden | GCF_000830375.1 |
| *Apilactobacillus kunkeei* LAan | Sweden | GCF_019575995.1 |
| *Apilactobacillus kunkeei* LAce | Sweden | GCF_001421115.1 |
| *Apilactobacillus kunkeei* LAfl | Sweden | GCF_001421135.1 |
| *Apilactobacillus kunkeei* LAko | Sweden | GCF_001281205.1 |
| *Apilactobacillus kunkeei* LAla | Sweden | GCF_001281215.1 |
| *Apilactobacillus kunkeei* LAni | Sweden | GCF_001281285.1 |
| *Apilactobacillus kunkeei* YH-15 | Sweden | GCF_001281265.1 |
| *Apilactobacillus kunkeei* LAdo | Sweden | GCF_001308205.1 |
| *Apilactobacillus kunkeei* LAnu | Sweden | GCF_001308215.1 |
| *Apilactobacillus kunkeei* LMbe | Sweden | GCF_001308185.1 |
| *Apilactobacillus kunkeei* LMbo | Sweden | GCF_001308195.1 |
| *Apilactobacillus kunkeei* MP2 | Cile | GCF_001314945.1 |
| *Apilactobacillus kunkeei* FF30-6 | Japan | GCF_001949975.2 |
| *Apilactobacillus kunkeei* UASWS1867-NN5 | Switzerland | GCF_005930915.1 |
| *Apilactobacillus kunkeei* UASWS1869-NN19 | Switzerland | GCF_005930925.1 |
| *Apilactobacillus kunkeei* UASWS1870-NN20 | Switzerland | GCF_005930935.1 |
| *Apilactobacillus kunkeei* UASWS1868-NN17 | Switzerland | GCF_005930975.1 |
| *Apilactobacillus kunkeei KA_01* | USA | GCF_006493415.1 |
| *Apilactobacillus kunkeei* 3L | USA | GCF_006493615.1 |
| *Apilactobacillus kunkeei* R13 | Germany | GCF_023218665.1 |
| *Apilactobacillus kunkeei* R5 | Germany | GCF_023218695.1 |
| *Apilactobacillus kunkeei 26* | Germany | GCF_023218705.1 |
| *Apilactobacillus kunkeei* 9 | Germany | GCF_023218755.1 |
| *Apilactobacillus kunkeei* 10 | Germany | GCF_023218775.1 |
| *Apilactobacillus kunkeei* 3 | Germany | GCF_023218815.1 |

**Supplementary Table 3.** Relative abundance (%) per microbial taxa and sample.

| | *Acinetobacter* | *Apilactobacillus* | *Arsenophonus* | *Bartonella* | *Bifidobacterium* | *Bombilactobacillus* | *Commensalibacter* | *Frischella* | *Gilliamella* | *Lactobacillus* | *Pseudomonas* | *Snodgrassella* |
|---|---|---|---|---|---|---|---|---|---|---|---|---|
| **BZ1** | 0.00 | 0.11 | 0.00 | 0.02 | 0.04 | 0.12 | 0.01 | 0.12 | 0.15 | 0.36 | 0.00 | 0.06 |
| **BZ2** | 0.00 | 0.11 | 0.00 | 0.01 | 0.05 | 0.19 | 0.01 | 0.16 | 0.14 | 0.30 | 0.00 | 0.03 |
| **BZ3** | 0.00 | 0.09 | 0.00 | 0.01 | 0.05 | 0.23 | 0.02 | 0.05 | 0.18 | 0.30 | 0.00 | 0.07 |
| **BZ4** | 0.00 | 0.09 | 0.00 | 0.02 | 0.07 | 0.12 | 0.02 | 0.15 | 0.16 | 0.29 | 0.00 | 0.09 |
| **BZ5** | 0.00 | 0.06 | 0.00 | 0.02 | 0.07 | 0.11 | 0.02 | 0.23 | 0.18 | 0.22 | 0.00 | 0.10 |
| **BZ6** | 0.00 | 0.05 | 0.00 | 0.03 | 0.06 | 0.16 | 0.01 | 0.08 | 0.23 | 0.32 | 0.00 | 0.06 |
| **BZ7** | 0.00 | 0.09 | 0.00 | 0.02 | 0.07 | 0.21 | 0.02 | 0.04 | 0.11 | 0.39 | 0.00 | 0.06 |
| **BZ8** | 0.00 | 0.07 | 0.00 | 0.01 | 0.03 | 0.19 | 0.00 | 0.07 | 0.16 | 0.38 | 0.00 | 0.11 |
| **BZ9** | 0.00 | 0.08 | 0.00 | 0.00 | 0.02 | 0.20 | 0.00 | 0.04 | 0.18 | 0.42 | 0.00 | 0.06 |
| **BZ10** | 0.00 | 0.06 | 0.00 | 0.01 | 0.08 | 0.30 | 0.00 | 0.07 | 0.17 | 0.22 | 0.00 | 0.09 |
| **CM1** | 0.04 | 0.02 | 0.00 | 0.19 | 0.10 | 0.10 | 0.04 | 0.00 | 0.14 | 0.14 | 0.00 | 0.24 |
| **CM2** | 0.02 | 0.04 | 0.01 | 0.38 | 0.01 | 0.00 | 0.41 | 0.08 | 0.05 | 0.01 | 0.00 | 0.01 |
| **CM3** | 0.18 | 0.01 | 0.00 | 0.18 | 0.04 | 0.03 | 0.03 | 0.26 | 0.16 | 0.04 | 0.04 | 0.02 |
| **CM4** | 0.00 | 0.00 | 0.00 | 0.64 | 0.05 | 0.01 | 0.00 | 0.01 | 0.08 | 0.02 | 0.18 | 0.01 |
| **CM5** | 0.00 | 0.01 | 0.00 | 0.25 | 0.02 | 0.00 | 0.09 | 0.14 | 0.17 | 0.04 | 0.15 | 0.12 |
| **CM6** | 0.03 | 0.12 | 0.00 | 0.40 | 0.03 | 0.00 | 0.05 | 0.06 | 0.16 | 0.04 | 0.00 | 0.10 |
| **CM7** | 0.00 | 0.08 | 0.00 | 0.48 | 0.02 | 0.01 | 0.18 | 0.12 | 0.08 | 0.02 | 0.00 | 0.01 |
| **CM8** | 0.00 | 0.03 | 0.00 | 0.56 | 0.04 | 0.00 | 0.25 | 0.00 | 0.07 | 0.03 | 0.00 | 0.02 |
| **CM9** | 0.00 | 0.01 | 0.00 | 0.05 | 0.05 | 0.00 | 0.02 | 0.05 | 0.26 | 0.20 | 0.02 | 0.32 |
| **CM10** | 0.00 | 0.09 | 0.00 | 0.31 | 0.15 | 0.05 | 0.12 | 0.02 | 0.11 | 0.08 | 0.00 | 0.06 |
| **GH1** | 0.00 | 0.04 | 0.00 | 0.36 | 0.13 | 0.09 | 0.02 | 0.00 | 0.12 | 0.20 | 0.00 | 0.04 |
| **GH2** | 0.00 | 0.02 | 0.01 | 0.49 | 0.05 | 0.03 | 0.01 | 0.00 | 0.13 | 0.12 | 0.02 | 0.12 |
| **GH3** | 0.00 | 0.05 | 0.00 | 0.44 | 0.07 | 0.09 | 0.02 | 0.00 | 0.17 | 0.12 | 0.00 | 0.03 |
| **GH4** | 0.01 | 0.03 | 0.03 | 0.76 | 0.04 | 0.03 | 0.01 | 0.00 | 0.04 | 0.03 | 0.00 | 0.02 |
| **GH5** | 0.00 | 0.04 | 0.00 | 0.44 | 0.07 | 0.02 | 0.07 | 0.01 | 0.05 | 0.05 | 0.16 | 0.09 |
| **GH6** | 0.00 | 0.04 | 0.00 | 0.70 | 0.05 | 0.01 | 0.06 | 0.03 | 0.07 | 0.03 | 0.00 | 0.00 |
| **GH8** | 0.00 | 0.07 | 0.00 | 0.72 | 0.02 | 0.01 | 0.05 | 0.01 | 0.03 | 0.06 | 0.00 | 0.02 |
| **GH9** | 0.00 | 0.04 | 0.00 | 0.27 | 0.03 | 0.00 | 0.15 | 0.04 | 0.35 | 0.05 | 0.05 | 0.02 |
| **GH10** | 0.00 | 0.03 | 0.00 | 0.07 | 0.15 | 0.04 | 0.05 | 0.03 | 0.12 | 0.22 | 0.00 | 0.28 |
| **SLS1** | 0.00 | 0.07 | 0.00 | 0.01 | 0.04 | 0.13 | 0.00 | 0.08 | 0.10 | 0.52 | 0.00 | 0.05 |
| **SLS2** | 0.00 | 0.03 | 0.00 | 0.01 | 0.04 | 0.20 | 0.00 | 0.01 | 0.10 | 0.54 | 0.00 | 0.06 |
| **SLS3** | 0.00 | 0.09 | 0.00 | 0.02 | 0.05 | 0.10 | 0.01 | 0.05 | 0.23 | 0.36 | 0.06 | 0.04 |
| **SLS4** | 0.00 | 0.09 | 0.00 | 0.00 | 0.04 | 0.20 | 0.00 | 0.05 | 0.07 | 0.50 | 0.00 | 0.04 |
| **SLS5** | 0.00 | 0.11 | 0.00 | 0.03 | 0.03 | 0.16 | 0.01 | 0.07 | 0.12 | 0.38 | 0.02 | 0.07 |
| **SLS6** | 0.00 | 0.06 | 0.00 | 0.01 | 0.05 | 0.18 | 0.01 | 0.02 | 0.11 | 0.50 | 0.00 | 0.06 |

| | | | | | | | | | | | | |
|---|---|---|---|---|---|---|---|---|---|---|---|---|
| **SLS7** | 0.00 | 0.09 | 0.00 | 0.00 | 0.03 | 0.13 | 0.00 | 0.06 | 0.09 | 0.56 | 0.00 | 0.03 |
| **SLS8** | 0.00 | 0.06 | 0.00 | 0.00 | 0.04 | 0.18 | 0.00 | 0.04 | 0.09 | 0.56 | 0.00 | 0.03 |
| **SLS9** | 0.00 | 0.09 | 0.00 | 0.01 | 0.04 | 0.22 | 0.00 | 0.05 | 0.18 | 0.37 | 0.00 | 0.04 |
| **SLS10** | 0.00 | 0.07 | 0.00 | 0.01 | 0.04 | 0.19 | 0.00 | 0.04 | 0.10 | 0.48 | 0.00 | 0.07 |
| **VS1** | 0.00 | 0.04 | 0.00 | 0.04 | 0.04 | 0.12 | 0.01 | 0.02 | 0.17 | 0.51 | 0.02 | 0.03 |
| **VS2** | 0.00 | 0.02 | 0.00 | 0.10 | 0.06 | 0.12 | 0.01 | 0.04 | 0.15 | 0.41 | 0.02 | 0.08 |
| **VS3** | 0.00 | 0.02 | 0.00 | 0.36 | 0.03 | 0.07 | 0.00 | 0.05 | 0.17 | 0.28 | 0.01 | 0.01 |
| **VS4** | 0.00 | 0.03 | 0.00 | 0.14 | 0.03 | 0.14 | 0.01 | 0.03 | 0.13 | 0.47 | 0.00 | 0.02 |
| **VS5** | 0.00 | 0.09 | 0.00 | 0.01 | 0.03 | 0.20 | 0.00 | 0.03 | 0.17 | 0.45 | 0.00 | 0.01 |
| **VS6** | 0.00 | 0.01 | 0.00 | 0.37 | 0.03 | 0.04 | 0.01 | 0.05 | 0.11 | 0.35 | 0.00 | 0.04 |
| **VS7** | 0.00 | 0.03 | 0.00 | 0.00 | 0.02 | 0.29 | 0.00 | 0.04 | 0.13 | 0.42 | 0.00 | 0.06 |
| **VS8** | 0.00 | 0.03 | 0.00 | 0.01 | 0.03 | 0.24 | 0.00 | 0.03 | 0.16 | 0.45 | 0.00 | 0.05 |
| **VS9** | 0.00 | 0.02 | 0.00 | 0.05 | 0.04 | 0.18 | 0.00 | 0.05 | 0.15 | 0.41 | 0.05 | 0.04 |
| **VS10** | 0.00 | 0.02 | 0.00 | 0.18 | 0.04 | 0.06 | 0.00 | 0.04 | 0.17 | 0.44 | 0.01 | 0.03 |
| **ZT1** | 0.00 | 0.14 | 0.03 | 0.19 | 0.04 | 0.00 | 0.20 | 0.00 | 0.14 | 0.05 | 0.13 | 0.07 |
| **ZT2** | 0.00 | 0.02 | 0.90 | 0.01 | 0.01 | 0.00 | 0.03 | 0.00 | 0.00 | 0.01 | 0.02 | 0.00 |
| **ZT3** | 0.00 | 0.05 | 0.27 | 0.34 | 0.08 | 0.03 | 0.04 | 0.00 | 0.08 | 0.08 | 0.00 | 0.02 |
| **ZT4** | 0.00 | 0.04 | 0.01 | 0.41 | 0.07 | 0.08 | 0.08 | 0.08 | 0.01 | 0.22 | 0.00 | 0.00 |
| **ZT5** | 0.00 | 0.18 | 0.00 | 0.01 | 0.02 | 0.08 | 0.03 | 0.00 | 0.00 | 0.04 | 0.00 | 0.65 |
| **ZT6** | 0.00 | 0.13 | 0.02 | 0.37 | 0.05 | 0.03 | 0.13 | 0.06 | 0.07 | 0.05 | 0.00 | 0.09 |
| **ZT7** | 0.00 | 0.11 | 0.00 | 0.33 | 0.14 | 0.07 | 0.13 | 0.08 | 0.04 | 0.03 | 0.00 | 0.07 |
| **ZT8** | 0.00 | 0.06 | 0.00 | 0.23 | 0.07 | 0.07 | 0.06 | 0.00 | 0.36 | 0.09 | 0.00 | 0.05 |
| **ZT9** | 0.00 | 0.22 | 0.08 | 0.31 | 0.06 | 0.01 | 0.21 | 0.00 | 0.01 | 0.04 | 0.00 | 0.05 |
| **ZT10** | 0.00 | 0.00 | 0.99 | 0.00 | 0.00 | 0.00 | 0.00 | 0.00 | 0.00 | 0.00 | 0.00 | 0.00 |

**Supplementary Table S4.** Microbial isolates (one per representative cluster in Fig.S15), identified with the sequencing of the 16S rRNA gene.

| Accession Number | Sequence ID/Strain | Identification |
|---|---|---|
| MG601163 | MT53 | *Lactobacillus melliventris* |
| MG601164 | MT1 | *Gilliamella apicola* |
| MG601165 | MT76 | *Lactobacillus apis* |
| MG601166 | MT61 | *Lactobacillus apis* |
| MG601167 | MT39 | *Apilactobacillus kunkeei* |
| MG601168 | MT11 | *Klebsiella oxytoca* |
| MG601169 | MT6 | *Gilliamella apicola* |
| MG601170 | GH1 | *Apilactobacillus kunkeei* |
| MG601171 | GH2 | *Apilactobacillus kunkeei* |
| MG601172 | GH3 | *Apilactobacillus kunkeei* |
| MT381710 | LGH12 | *Apilactobacillus kunkeei* |
| MT381711 | LGH13 | *Apilactobacillus kunkeei* |
| MT381712 | LGH15 | *Apilactobacillus kunkeei* |
| MT381713 | LGH34 | *Apilactobacillus kunkeei* |
| MT381714 | LGH35 | *Apilactobacillus kunkeei* |
| MT381715 | LGH43 | *Apilactobacillus kunkeei* |
| MT381716 | LGH49 | *Apilactobacillus kunkeei* |
| MT381717 | LGH53 | *Apilactobacillus kunkeei* |
| MT381718 | LGH55 | *Apilactobacillus kunkeei* |
| MT381719 | LGH63 | *Apilactobacillus kunkeei* |
| MT381720 | LGH70 | *Apilactobacillus kunkeei* |
| MT381721 | LU2 | *Gilliamella apicola* |
| MT381722 | LZ15 | *Apilactobacillus kunkeei* |
| MT381723 | LZ25 | *Apilactobacillus kunkeei* |
| MT381724 | LZ47 | *Apilactobacillus kunkeei* |
| MT381725 | LZ43 | *Apilactobacillus kunkeei* |
| MT381726 | LU5 | *Apilactobacillus kunkeei* |
| MT381727 | LZ1 | *Apilactobacillus kunkeei* |
| MT381728 | LZ4 | *Apilactobacillus kunkeei* |
| MT381729 | LZ28 | *Apilactobacillus kunkeei* |
| MT381730 | LZ48 | *Gilliamella apicola* |
| MT381731 | LU6 | *Gilliamella apicola* |
| MT381732 | LU12 | *Klebsiella oxytoca* |
| MT381733 | LGH4 | *Apilactobacillus kunkeei* |
| MT381734 | LGH5 | *Apilactobacillus kunkeei* |
| MT381735 | LGH6 | *Apilactobacillus kunkeei* |
| MT381736 | LZ32 | *Apilactobacillus kunkeei* |
